# Deepening into Intracellular Signaling Landscape through Integrative Spatial Proteomics and Transcriptomics in a Lymphoma Model

**DOI:** 10.3390/biom11121776

**Published:** 2021-11-26

**Authors:** Alicia Landeira-Viñuela, Paula Díez, Pablo Juanes-Velasco, Quentin Lécrevisse, Alberto Orfao, Javier De Las Rivas, Manuel Fuentes

**Affiliations:** 1 Department of Medicine and General Cytometry Service-Nucleus, USAL/IBSAL, 37000 Salamanca, Spain; alavi29@usal.es (A.L.-V.); pauladg@usal.es (P.D.); pablojuanesvelasco@usal.es (P.J.-V.); quentin@usal.es (Q.L.); orfao@usal.es (A.O.); 2 Proteomics Unit, Cancer Research Centre (IBMCC/CSIC/USAL/IBSAL), 37007 Salamanca, Spain; 3 Bioinformatics and Functional Genomics, Cancer Research Centre (IBMCC/CSIC/USAL/IBSAL), 37007 Salamanca, Spain; jrivas@usal.es

**Keywords:** affinity-based proteomics, human proteome project, LC-MS/MS, transcriptomics, size-exclusion-chromatography (SEC)

## Abstract

Human Proteome Project (HPP) presents a systematic characterization of the protein landscape under different conditions using several complementary-omic techniques (LC-MS/MS proteomics, affinity proteomics, transcriptomics, etc.). In the present study, using a B-cell lymphoma cell line as a model, comprehensive integration of RNA-Seq transcriptomics, MS/MS, and antibody-based affinity proteomics (combined with size-exclusion chromatography) (SEC-MAP) were performed to uncover correlations that could provide insights into protein dynamics at the intracellular level. Here, 5672 unique proteins were systematically identified by MS/MS analysis and subcellular protein extraction strategies (neXtProt release 2020-21, MS/MS data are available via ProteomeXchange with identifier PXD003939). Moreover, RNA deep sequencing analysis of this lymphoma B-cell line identified 19,518 expressed genes and 5707 protein coding genes (mapped to neXtProt). Among these data sets, 162 relevant proteins (targeted by 206 antibodies) were systematically analyzed by the SEC-MAP approach, providing information about PTMs, isoforms, protein complexes, and subcellular localization. Finally, a bioinformatic pipeline has been designed and developed for orthogonal integration of these high-content proteomics and transcriptomics datasets, which might be useful for comprehensive and global characterization of intracellular protein profiles.

## 1. Introduction

Increased understanding of the events taking place during intracellular signaling has revealed a highly dynamic protein landscape for eukaryotic cells, especially in pathological settings (such as cancer, neurodegenerative, auto-immune diseases, etc.,) [1,2,3]. Most of the subcellular signaling pathways are led by protein complexes, which are constantly being formed and resolved. Furthermore, proteins are shuttling between different subcellular localizations to execute the expected and/or programmed biological processes according to the needs and requirements of the cell. These fluctuations could produce re-wiring of signaling networks for enabling phenotypic changes required for the adaptation to microenvironmental or external perturbations and/or stimuli [4,5,6]. Given these temporal and spatial variabilities, high-throughput biochemical methods are required to deepen the knowledge on the interacting biomolecules across subcellular localizations during the response to external stimulus, drug administration, and drug resistance mechanisms, etc., [1,2,4,6,7].

Bearing this in mind, the recent advances in next-generation sequencing (NGS) and high-resolution mass spectrometry (MS/MS) have opened up strategies methodologically which provide novel insights useful for understanding the complexity of intracellular processes [8,9]. Among other parameters in protein functionality, subcellular localization is one of the main determinants for a particular protein’s intracellular dynamics and the final function; however, a global view of subcellular proteome organization remains relatively unknown, and it is currently studied by multipronged proteomics approaches. Despite some multiple integrations that have been recently performed (based on MS/MS and RNA-Seq, among others), there remains a huge interest in revealing the presence of proteoforms, multiprotein complexes, changes in transcript-protein, etc., [10,11,12,13]. Historically, subcellular localization of proteins has been determined by conventional biochemical approaches (mostly by targeting individual proteins); e.g., cell fractionation coupled with Western blot. Although additional development of large-scale GFP fusion protein-based [14], antibody-based assays [15,16], protein-metabolite interactions [17], Sequential Window Acquisition of All Theoretical Mass Spectra (SWATH-MS) characterization [18] has increased our knowledge of protein subcellular localization, these methods are labor-intensive, making them highly challenging and difficult to implement for high-content characterization in large cohort studies.

The development of mass spectrometry (MS/MS)-based proteomics coupled with protein extraction at subcellular level [19] as well as the high-throughput immunoassays coupled with size-exclusion chromatography (SEC) [20] have opened up new possibilities to query the spatial intracellular organization of the proteome on a larger scale [21]. More recently, the Aebersold group [22] has developed a strategy combining mass-spectrometry and SEC. Other similar attempts involve machine learning algorithms to assign subcellular localization based on protein quantification across multiple subcellular fractions by MS/MS. However, all these studies provide proteome-wide information but lack the multiple combinations between several proteomics characterizations and comprehensive integration with transcriptomics.

This study has two main purposes: i. Evaluation of protein extraction or complementary characterization with MS/MS-based proteomics and affinity proteomics (SEC-MAP), which may be useful for bioinformatic orthogonal integration with deep transcriptomics characterization; ii. generation of systematic pipeline for comprehensive orthogonal integration of protein subcellular localization, affinity proteomics (SEC-MAP), MS/MS data sets, and RNA-seq information in human lymphoma cell line, like a model for mapping cancer protein interactions. Moreover, the combination of these methodologies helps to deepen the knowledge about the architecture of the cells and the complexity of the spatial organization of the proteome, which can be a tool for interpreting a multiscale map of protein systems—which is relevant for deciphering cell signaling pathways linked to genetic perturbations/alterations, therapeutic interventions, or another external stimulus.

## 2. Materials and Methods

### 2.1. Cell Cultures

Human Ramos cell line (Burkitt′s lymphoma, European Collection of Authenticated Cell Cultures—ECACC-Cat. no: 85030802) was cultured in RPMI 1640 medium supplemented with L-glutamine (Gibco, Whaltham, MA, USA), 10% (*v*/*v*) fetal bovine serum (Gibco, Whaltham, MA, USA) and 1% (*v*/*v*) penicillin/streptavidin (Gibco, Whaltham, MA, USA). The growth was monitored daily and cells were incubated at 37 °C/5% CO_2_ with media renewal every 2–3 days.

### 2.2. Protein Extraction and Quantification

For protein extraction, 40 × 10^6^ Ramos cells were pelleted by centrifugation and washed three times with PBS, and further centrifuged for 5 min (min.) at 1200 rpm. All the different protein extraction buffers were supplemented with phosphatase and protease inhibitors: tris [2-carboxyethyl]phosphine] (TCEP—Sigma-Aldrich, Burlington, MA, USA) 1 mM, phenylmethylsulfonyl fluoride (PMSF—Sigma-Aldrich, USA) 1 mM, sodium fluoride (NaF—Sigma-Aldrich, USA) 1 mM, sodium orthovanadate (Sigma-Aldrich, USA) 1 mM, β-glycerophosphate (Sigma-Aldrich, USA) 1 mM and sodium pyrophosphate tetrabasic decahydrate (Sigma-Aldrich, USA) 1 mM. All centrifugation and incubation steps were carried out at 4 °C. The extracted proteins from each strategy were stored at −20 °C until further use.

Protein quantification was performed by Coomassie Plus (Bradford) Assay Kit (ThermoFisher Scientific, Cat. no: 23236, Waltham, MA, USA) and Pierce™ BCA Protein Assay Kit (ThermoFisher Scientific, Cat. no: 23225, USA), following the manufacturer’s instructions. Each sample (20 µg) was separated in 12% SDS-PAGE gel under reducing conditions. Subsequently, SDS-PAGE gels were stained with 0.1% (*w*/*v*) Coomassie brilliant blue and silver stains solutions according to standard laboratory procedure [23,24,25].

The different protein extraction procedures, which are evaluated herein, are described below and summarized in Table 1.

#### 2.2.1. Protocol #1

Total of 375 µL of lysis buffer (140 mM NaCl, 50 mM EDTA, 10% (*v*/*v*) glycerol, 1% octylphenoxy poly(ethyleneoxy)ethanol—IGEPAL-, 20 mM Tris-HCl pH = 7 supplemented with proteases and phosphates inhibitors) per 1 × 10^7^ cells was added, and cells were incubated on ice for 15 min. After that, the sample was centrifuged for 15 min at 15,000× *g* and the supernatant containing the protein content was stored at −20 °C until further analysis.

#### 2.2.2. Protocol #2

As protocol #1, 1 × 10^7^ cells were incubated with 375 µL of lysis buffer (20 mM HEPES pH = 8 and 9 M urea). Cell lysis was performed by sonication on ice (3 times 5 s bursts and 1 min break). Afterward, it was centrifuged for 15 min at 15,000× *g* and the supernatant containing the total protein content was stored at −20 °C until further analysis.

#### 2.2.3. Protocol #3

Similar procedure as protocol #2 with a lysis buffer containing 7 M urea, 2 M thiourea, and 30 mM Tris-HCl pH = 8.5. The sample was then centrifuged for 15 min at 12,000× *g* and the supernatant was stored at −20 °C until further analysis.

#### 2.2.4. Protocol #4

The same procedure as protocol #3 but with a different lysis buffer: 5 mM HEPES pH 8, 10 mM MgCl_2_, 140 mM NaCl and 0.01% Tween 20.

#### 2.2.5. Protocol #5

Total of 1 × 10^7^ cells were incubated with 375 µL of hypotonic buffer (30 mM HEPES pH = 8, 15 mM KCl, 2 mM MgCl_2_, 1 mM EDTA and 20% glycerol) supplemented with 10% (*v*/*v*) laurylmaltoside (n-Dodecyl-β-D-maltoside) for 30 min in rotation at 4 °C. Afterward centrifugation at 16,000× *g* for 5 min was carried out and the supernatant containing the proteins was collected.

#### 2.2.6. Protocol #6

Similar to protocol #5 with a difference that the lysis buffer was supplemented with 1.5% (*v*/*v*) Triton X-100 instead of laurylmaltoside.

#### 2.2.7. Protocol #7

In this case, 1 × 10^7^ cells were incubated with 375 µL of buffer (5 mM HEPES pH 8, 10 mM MgCl_2_, 140 mM NaCl and 0.01% Tween 20) and it was centrifuged for 15 min at 13,000× *g*. After centrifugation, the supernatant enrichment in cytoplasmic (Cyt) proteins was collected and the pellet was re-suspended in 375 µL of buffer supplemented with 38% (*v*/*v*) octyl-β-D-glucopyranoside. The sample was sonicated on ice (3 times 3 s bursts and 1 min break). After sonication, it was incubated for 30 min on ice and subsequently, it was centrifuged for 15 min at 13,000× *g*. Finally, the supernatant enriched in membrane proteins (Mem) was collected [26].

#### 2.2.8. Protocol #8

Ramos cell pellets were re-suspended at a volume equal to 5 times that of the cell pellet in hypotonic buffer supplemented with protease and phosphatase inhibitors and 0.015% digitonin following the methodology published by Díez P. et al., 2015 [19].

Then, it was incubated on a rotatory shaker for 30 min and then it was centrifuged for 5 min at 500× *g*. After centrifugation, cytoplasmic (Cyt) proteins were found in the resulting supernatant 1. The pellet 1 was washed 3× with hypotonic buffer and it was centrifuged for 5 min at 500× *g* at 4 °C. The following fractions were processed similarly in a stepwise manner. For the supernatant 2 containing organelle (Org) proteins, a hypotonic buffer was supplemented with 0.5% Tween 20; for the supernatant 3 containing nuclear (Nuc) proteins, a hypotonic buffer supplemented with 14 mM NaCl was used; for the supernatant 4 containing membrane (Mem) proteins, a hypotonic buffer supplemented with 1% laurylmaltoside was used.

#### 2.2.9. Protocol #9

Similar procedure as protocol #8 for cytoplasmic and organelle subcellular fractions. The membrane fraction was extracted with a hypotonic buffer supplemented with 0.5% octylphenoxy poly(ethyleneoxy)ethanol (IGEPAL) and centrifuged for 5 min at 3000× *g*. A hypotonic buffer supplemented with 1% laurylmaltoside was used for the extraction of the nuclear fraction.

### 2.3. Proteomics Analysis

#### 2.3.1. Protein Digestion and LC-MS/MS Analysis

Each lane in SDS-PAGE gel (loaded with 15 µg protein extract) was cut into five equal fragments and digested using the method described by Olsen et al. with slight modifications (each piece was destained with 15 mM potassium ferrocyanide and for reduction and 50 mM sodium thiosulfate was used for the alkylation process) and incubated with 10 mM DTT at 56 °C for 45 min and then 55 mM iodoacetamide (IAA) was added and incubated at room temperature (RT) for 30 min, respectively. Trypsin (6.25 ng/mL) at 37 °C for 18 h was used for protein digestion, and the peptide solution was acidified with formic acid (FA) and desalted using C18-Stage-Tips columns) [19,27]. Samples were partially dried and stored at −20 °C until analyzed by LC-MS/MS.

A nanoUPLC system (nanoAcquity, Waters Corp., Milford, MA, USA) coupled to an LTQ-Velos-Orbitrap mass spectrometer (Thermo Fisher Scientific, San Jose, CA, USA) via a nanoelectrospray ion source (NanoSpray flex, Proxeon, Thermo) was used to study with LC-MS/MS. Peptides dissolution was carried out using 0.5% FA/3% acetonitrile (ACN). A trapping column (nanoACQUITY UPLC 2G-V/M Trap Symmetry 5 μm particle size, 180 μm × 20 mm C18 column, Waters Corp., Milford, MA, USA) was used to load. Separation was made with a linear gradient from 7% to 35% solvent B (ACN/0.1% FA) at a flow rate of 250 nL/min over 120 min in a nanoACQUITY UPLC BEH 1.7 μm, 130 Å, 75 μm × 250 mm C18 column (Waters Corp., Milford, MA, USA) [19,26,27]. Tandem mass spectra (MS/MS) acquisition and survey MS scan were applied to a data-dependent automatic switch using the positive ion mode of the nUPLC-LTQ-Orbitrap Velos. Acquisition scan was made with lock mass option enabled for the 445.120025 ion and mass range of m/z 400 to 1600. In the ion trap for fragmentation by collision-induced dissociation with 35% normalized energy, 10 ms activation time, q = 0.25, ± 2 m/z precursor isolation width and wideband activation, we selected the 20 peaks with the most intensity and with ≥2 charge state and above the 500 intensity threshold. Automatic gain control was 1 × 10^6^ for MS and 5 × 10^3^ for MS/MS scans and dynamic exclusion was enabled for 90 s [19,26,27]. 

#### 2.3.2. Database Search

Raw data were converted into Mascot general file (.mgf) and target-decoy strategy was used to search in the neXtProt database (release 2016-02). Comet version (v.) 2015.01 rev.2 [28] was used to identify the peak list (.mgf file) obtained from MS/MS spectra and SearchGUI v. 1.30.1 [29] was used.

Concatenated target/decoy [30] version of the human complement of neXtProt release 2016-02 (41,992 sequences) was used for protein identification. SearchGUI v. 1.30.1 was used to design decoy sequences (the reverse target sequences) [29]. The process for the identification of the settings was as follows: trypsin with a maximum of two missed cleavages; 10.0 ppm as MS1 and 0.5 Da as MS2 tolerances; fixed modifications: carbamidomethylation of cysteine (+57.021464 Da). The variable modifications were acetylation of protein n-terminus (+42.010565 Da) and oxidation of methionine (+15.994915 Da) [19,26,27,28,29,30].

PeptideShaker v. 0.41.1 [31] was used to infer peptides and proteins from the identified spectra. A method, as the one published by our group in 2015, was used for the validation of decoy hit distribution (at a 1.0% False Discovery Rate—FDR—for Peptide Spectrum Matches –PSMs-, peptides and proteins) [19]. It is obtained at protein/peptide/PSM levels, the FDR (%), true positives, and false positives values for each replicated (1, 2, 3): Cyt 1 (0.99, 2899, 29/0.98, 10474, 104/1.0, 17627, 178); Cyt 2 (1.0, 3183, 32/1.0, 12897, 130/1.0, 20147, 203); Cyt 3 (0.99, 2802, 28/0,99, 10857, 109/1.0, 5666, 57); Mem 1 (0.93, 1166, 11/0.97, 2651, 26/0.99, 1303, 13); Mem 2 (0.97, 1122, 11/0.97, 1937, 19/0.99, 1607, 16); Mem 3 (1.0, 893, 9/0.98, 1523, 15/1.0, 1291, 13); Org 1 (0.97, 2543, 25/0.99, 7503, 75/0.99, 10305, 103); Org 2 (0.99, 2294, 23/0.99, 6919, 69/0.98, 4933, 49); Org 3 (0.96, 2465, 24/0.98, 7045, 70/1.0, 10507, 106); Nuc 1 (0.99, 2498, 25/0.98, 7145, 71/0.97, 915, 9); Nuc 2 (0.96, 2577, 25/1.0, 8938, 90/1.0, 4469, 45); Nuc 3 (1.0, 2872, 29/0.99, 10937, 109/0.99, 4585, 46). The protein-level FDR is an estimate and not all proteins that exceeded the threshold were “confidently identified”.

The mass spectrometry data and the identification results have been deposited to the ProteomeXchange Consortium [32] via the PRIDE partner repository [33] with the dataset identifier PXD003939 and 10.6019/PXD003939. The data can be accessed with the following credentials upon login to the PRIDE website (http://www.ebi.ac.uk/pride/archive/login, accessed on 30 March 2021): Username: reviewer85106@ebi.ac.uk, Password: 0fVloZfQ.

#### 2.3.3. Quantitative Analysis of MS/MS Datasets

Raw data were analyzed as reported by Paula et al. in 2021 [34]: i. For expression level measuring, the Label-Free Quantification method MaxLFQ [35] with the MaxQuant Suite v. 1.5.3.30 was used [36]. ii. PTXQC package v. 0.80.1 [37] in R v. 3.2.4 [38] was utilized for quality control analysis. iii. Perseus framework v. 1.5.3.2 was performed for ulterior analysis. Additionally, for each subcellular fraction (Mem, Cyt, Org and Nuc) total proteins and exclusive proteins were determined.

#### 2.3.4. RNA-Sequencing Transcriptomics

RNA-Seq data from Ramos B-cell line was obtained with Illumina Genome Analyzer IIx with paired layout (experiment SRX105534: http://www.ncbi.nlm.nih.gov/sra/SRX105534, accessed on 30 March 2019 taken from the study SRP00931 (http://trace.ncbi.nlm.nih.gov/Traces/sra/?study=SRP009316, accessed on 30 March 2019) [39] from SRA (Sequence Read Archive) database. Gene expression analysis consisted of calculated values of FPKM fragment per kilobase of exon per million fragments mapped) on base for each gene on the following steps [19]: i. Use of SRA tools [40] to obtain the SRR387395 dataset from SRA database [40] and the subsequent conversion of the SRA file to paired-end fastq files; ii. trimming of the data with Trimmomatic [41]; iii. use the program STAR [42] to align the reads to ENSEMBL GRCh37 genome; iv. generation of a binary sequence alignment map (BAM) with SAMtools [43]; v. calculating the FPKM value for each gene with CuffLinks from the BAM files [44]; vi.-Use of the neXtProt ID mapping table to map ENSG_IDs within the neXtProt database v. 2016-02 (ftp://ftp.nextprot.org/pub/current_release/mapping/, accessed on 30 March 2019). Finally, a total of 19,518 neXtProt IDs could be mapped within the RNA-Seq dataset, out of this 9523 neXtProt IDs had FPKM > 1.

### 2.4. Biotin Protein Labeling

Protein extracts obtained from protocols #1, #4, #5, #6, #7, #8, and #9 were biotin-labelled following the procedure described by Häggmark A. et al. 2013 [45]. All the cell lysates were incubated with two different concentrations (10 µg/µL—protocol A—and 1 µg/µL—protocol B-) of NHS-PEG_4_-biotin (Thermo Scientific, no: 21363, USA) for 2 h at 4 °C. Biotin-labeling reactions were stopped with 4.5 µL for protocol A and 50% of the volume of the biotin-labeling solution for concentration for protocol B of 0.5 M Tris-HCl pH = 8.

After that, Amicon^®^ Ultra-0.5 centrifugal filter 3K (Millipore-Merk, Damrstad, Germany) was used for removing the excess of biotin until a final volume of 110 µL (maximum volume of injection in column HPLC). In both approaches, 75 µg of protein was conjugated with biotin.

### 2.5. Size Exclusion Chromatography (SEC): Fast Protein Liquid Chromatography (FPLC)

Equipment HPLC 1100 series (Agilent) and column Superdex^®^ 200 Increase 10/300 GL (GE Healthcare, Illinois, Sigma-Aldrich, USA) was used for protein fractionation based on molecular weight (MW). As MW standards, a mix of five purified proteins (Ferritin—440 kDa, aldolase—158 kDa-, conalbumin—75 kDa, ovalbumin—43 kDa and ribonuclease A—13.7 kDa) was used. Each sample was fractionated and collected in 24 aliquots at 0.5 mL/min. (flow rate) and PBS 1X Na^2+^/K^+^- tween 20 0.5% (*v*/*v*) as running buffer.

Then, all 24 collected fractions were merged in 8 fractions within defined MW ranges (fraction 1—166–473 kDa, fraction 2—121–142 kDa, fraction 3—74–103 kDa, fraction 4—54–63 kDa, fraction 5—33–46 kDa, fraction 6—24–28 kDa, fraction 7—17–21 kDa and fraction 8—11–15 kDa) by using Amicon^®^ Ultra-0.5 centrifugal filter 3K (Millipore-Merk, Germany) until a final volume of 100 µL. All steps were performed at 4 °C. Each collected fraction was stored at −20 °C until incubation for protein microarray.

### 2.6. Protein Microarrays

Microarray preparation and performance evaluation were done following the procedures previously described by Sierra-Sánchez et al., 2017 [46]. The glass slide surface was activated by incubation with 2% (*v*/*v*) 3-(2-Aminoethylamino) propyldimethoxymethyl (MANAE) silane in acetone for 30 min and slight shaking at RT [47]. Then, activated glass slides were washed with acetone and Milli-Q water and dried with compressed filtered air. For antibody array printing, a non-contact inkjet printing technology (Arrayjet Inc., Edinburg, UK) was employed with a slide-out of 7 similar subarrays and 5 replicates per sample. The array content is described in Table S1 where 205 antibodies targeting 162 proteins, among positive and negative controls, were included (Table S2). All the samples were prepared at 1:1 (*v*/*v*) dilution with JetStar™ (Arrayjet Inc., Edingburgh, UK) printing buffer C.

### 2.7. Evaluation of Array Performance at Different MW Fractions

Protein microarrays were blocked with blocking solution PBS and 1% blocker BSA—10X, (Thermo Fisher Scientific, USA) for 1 h at RT with mild stirring. Then, blocked protein microarrays were thoroughly washed with distilled water. Regarding sample handling, 50% volume of each 24 collected fractions was processed with epitope retrieval treatment (30 min at 56 °C and 1 min at 20 °C) and at last, the remaining 50% samples were combined in one single solution. After that, 100 µL per subarray of each mix were incubated at 4 °C, with mild stirring. After overnight incubation, 100 µL of Cy3- Streptavidin (1:200 -*v*/*v*-) was added to each subarray, and it was incubated for 1 h in darkness in a humidified chamber at RT. Finally, the protein microarrays were washed and dried for further acquisition of array images. All steps were performed at RT unless otherwise specified.

### 2.8. Image Analysis and Data Acquisition

TIFF images obtained at different exposition times to achieve optimal images with SensoSpot^®^ Fluorescence Microarray (Sensovation Gmbh, Radolfzell, Germany) were analyzed using GenePix Pro v. 6.0 software. Parameters were set to quantify light intensity values at Cy3 (λ = 532 nm) emission wavelength.

### 2.9. Protein Microarray Data Processing

Signal intensity values were processed by performing background subtraction, filtering, and housekeeping processes.

#### Normalization

To remove the background, the following was used:

$$\tau_{{\mathrm{iMM}}_{n}}=\left(\tilde{\delta }-k\right)-2\sigma k$$

where:

$\tau_{{\mathrm{iMM}}_{n}}$: signal intensity value spot i containing MasterMix (MM_n_).

$\tilde{\delta }$: median intensity value per spot.

k: constant of subarray background intensity.

2σk: variance of signal intensity background per subarray.

To remove the background effect of the mastermix (MM), as the same MM for each antibody was used, the following equation was used:

$$S_{i}=\tau_{{\mathrm{iMM}}_{n}}-\left({\tilde{\delta }}_{{\mathrm{MM}}_{n_{\max}}}+{\tilde{\delta }}_{{\mathrm{MM}}_{n_{\max}}}\cdot 0.05\right)$$

where:

$S_{i}$: signal intensity value of spot i without background.

${\tilde{\delta }}_{{\mathrm{MM}}_{n_{\max}}}$: maximum value of median intensity MM.

After background subtraction, the signal was normalized against a positive control (biotin). For further analysis, only the proteins detected with >50% of spotted antibodies displaying a normalized signal > 0 were included.

### 2.10. SEC-MAP Database

To combine the SEC-MAP data sets with transcriptomics and other proteomics characterization (LC-MS/MS), a database was designed and developed containing: i. Protein ratio at SEC-MAP for each MW fraction and protein extraction procedure; ii. Antibody info: type (monoclonal/polyclonal), supplier, and developed; iii. Protein ID, NextProt ID, Uniprot ID, MW (expected/observed/theoretical, etc.,), subcellular localization; iv. Detection by LC-MS/MS previously reported in the cell type of interest [19]. 

### 2.11. Integration of Transcriptomics, Proteomics, and SEC-MAP Datasets

A database called “complete protein mapping” was designed and developed from reported LC-MS/MS characterization of Ramos cell line in our previously reported studies [19]. Total of 5672 proteins were detected by unique tryptic peptides in three technical replicates and the subcellular localization (Cyt, Mem, Org, Nuc) was included (Table S3). The genes coding for each of the detected proteins were mapped to chromosomes with the R-package BiomaRt [48]. neXtProt IDs are used to merge the different datasets (RNA-Seq, LC-MS/MS, and SEC-MAP). In the case of proteins with more than one gene IDs, the gene ID with the highest FPKM value was selected, Table S4. In this study, a protein was considered to be fully observed if: i. Number of peptides ≥ 1; ii. FPKM ≥ 1; or iii. QAS value ≥ 1. Biological function analysis of observed proteins is based on DAVID and GeneTerm-Linker tools [49,50], which were used for functional enrichment analysis (FEA). Databases selected to find genes with annotated enriched terms were: (i) Gene Ontology (GO) using GO_BP, GO_CC and GO_MF; (ii) KEGG_PATHWAY; and (iii) the INTERPRO protein structural domain databases were used for generation of functional enrichment analysis (FEA). Moreover, a platform STING v. 11.0 [51] for the protein interactions obtained from SEC-MAP was used. For signaling pathways observed from the integration of proteomics and transcriptomics datasets, KEGG v. 93.0 [52] and Reactome v. 71 were used [53].

### 2.12. Visualization of Transcriptomics, Proteomics, and SEC-MAP Datasets

In this study, the software called Infinicyt™ 2.0 (Cytognos SL, Salamanca, Spain) has been employed for the visualization of multidimensional and multiparametric data sets. For that, a classification of each immunoassay was based on expected and theoretical subcellular localization and its correlation with the observed subcellular localization by the LC-MS/MS characterization. Hence, proteins were classified—according to subcellular localization—by positive selection in the following order: Nuc, Cyt, Mem, Org, Nuc-Org, Nuc-Cyt, Nuc-Mem, Org-Cyt, Org-Mem, Cyt-Mem, Nuc-Org-Cyt, Nuc-Org-Mem, Nuc-Cyt-Mem, Org-Cyt-Mem, and Nuc- Org-Cyt-Mem. Additionally, for each classified group, proteomics and transcriptomics integration were represented by APS, t-SNE plot, population burst, 2D (file number—*y*-axis- and each subcellular localization—*x*-axis-, and LFQ logarithmic—*y*-axis- and FPKM logarithmic—*x*-axis-). All these graphs (including 3D, tables, etc.,) are reported in Supplementary Materials Dataset I.

After that, a second classification was established according to proteomics values vs. transcriptomics values, represented by Log LFQ vs. Log FPKM, depicted in a 2D plot which allowed for classifying (according to proteomics vs. transcriptomics) five additional groups named: average population, average population, low Log LFQ vs. progressive Log FPKM, high Log LFQ vs. low Log FPKM, high Log LFQ vs. Log FPKM, and a group of outliers. Then, each group of proteins according to the subcellular localization was re-classified on these additional groups.

## 3. Results

**Figure 1:**
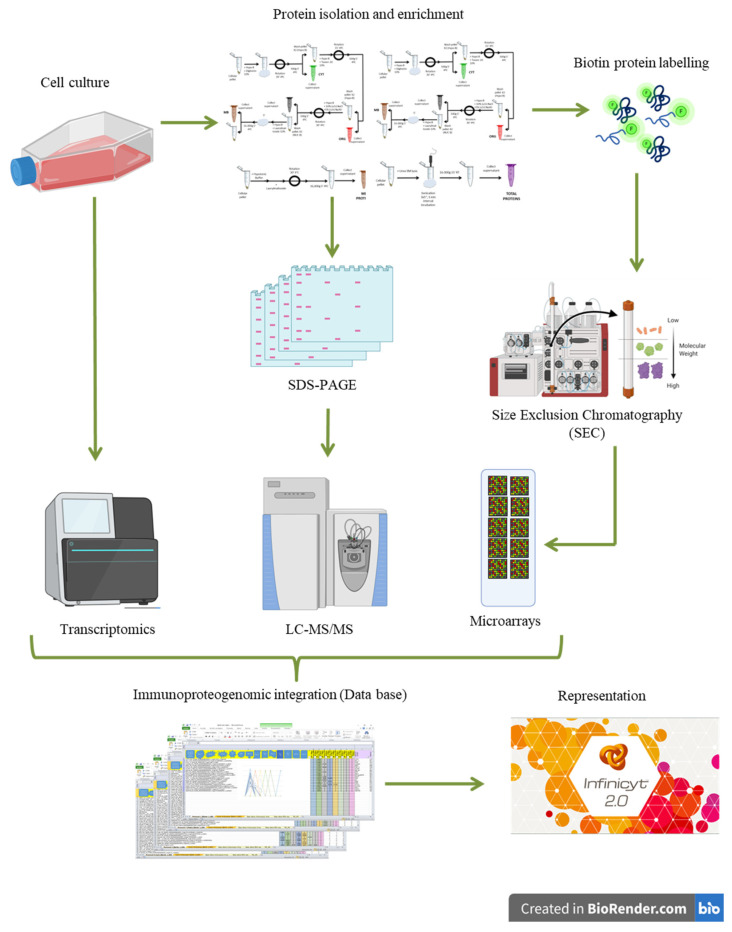
Schematic Representation of Experimental Design. Description of experimental steps involved for multi-omics integration.

In Figure 1, an overall representation of the experimental workflow performed for the systematic and multipronged multi-omics characterization and the multi-dimensional bioinformatics integration is shown.

### 3.1. Protein Extraction Strategies for Multi-Pronged Proteomics Characterization

Knowing and exploring the different cell lysis strategies is fundamental to know the compatibility of the different proteomics methodologies and the feasibility to perform an integration of multi-pronged proteomic strategies (LC-MS/MS and SEC-MAP, for example) (Figure 1). For that reason, this study compares nine different protein extraction methods (as described in materials and methods section -M&M-) on a cell line of interest (Ramos cell line, Burkitt’s lymphoma, RA1—ATTC: CRL-1596-). Each method is different regarding the chemical composition (Table 1); therefore, to allow a better understanding and comparison between them, they are named from #1–#9. In addition, protocols #7–#9 allow protein separation at different subcellular localizations. Then, all the protein extraction strategies—and subsequent chemical biotin labeling—are evaluated for SEC-MAP performance, according to: i. Efficiency of protein extraction: direct correlation with protein abundance (Figure S1) and expected subcellular localization. ii. Compatibility with protein labeling: biotin is commonly used in protein microarrays; however, several chemical components of the lysate buffers can cause interferences in the biotin conjugation protocol and array performance must be evaluated under these conditions (Figure S2). iii. SEC: to correctly analyze multi-protein complexes, the array performance is evaluated at several MW fractions to confirm that protein abundance is not affected by protein size (MW) and subcellular localization.

#### Performance of SEC-MAP: Effect of Protein Extraction Procedures and Biotin Conjugation

The total amount of protein extracted with each of the strategies show wide variability. For the protein total extraction protocols, the amount of protein obtained ranged between 34 and 61 µg/protein total per 10^6^ cells obtained, protocol #2 and #4, respectively. In the case of subcellular enrichment, the amount with membrane extraction protocol ranged from 169 to 270 µg/protein total per 10^6^ cells with protocol #5 and #6; and subcellular fraction protocols (#9 & #7), the range is between 82 and 166 µg/protein total per 10^6^ cells, respectively. These results confirm that the properties of chemical reagents—presented in the extraction buffers—are critical for the relative and/or absolute determination of protein abundance; but, it seems that protein distribution is not influenced as they remain comparable among all the protocols (Figure S1B).

About biotin conjugation, it does not show differences in all the analyzed MW by SEC (fraction 1—142–437 kDa –, fraction 2—74–121 kDa –, fraction 3—46–63 kDa –, fraction 4—26–39 kDa –, fraction 5—11–24 kDa). However, for antibody array performance, biotin-conjugated proteins present differences after SEC analysis (Figure S3). These are directly related to the effect on epitope recognition caused by chemical modification during biotin labeling. For SEC-MAP performance, it is also relevant that optimal conditions for SEC which were studied in all protein extractions in eight pre-defined MW ranges in the same antibody microarray. First, the number of identified proteins different is considered a parameter for comparison across the studied protein extraction strategies (Table S5). In this case, protocol #1 presents the highest number of identified total proteins, and protocol #6 the lowest. It is due to different detergent compositions in each protein extraction buffer (Figure S4A).

Moreover, a comparison of the observed subcellular localization of the proteins is made for each extraction procedure. A higher number of identified proteins are reached by protocol #1 and the lowest one by protocol #4. When the comparison is focused only on membrane proteins, protocol #5 displayed a higher number of identified proteins than protocol #6 (Table S6, Figure S4A). Likewise, protocols #8 and #9 for subcellular protein extractions (Cyt, Mem, Nuc, Org) gave similar numbers of identified proteins in all studied subcellular localizations (Table S6, Figure S4). As proof of concept, the SEC-MAP analysis focuses on BID, CASP 7, CASP 8, LYN, SYK, BTK, CDKN1B, DNAJB1, HSPD1, HSP90AA1, HIST1H4A, BAX, BAK1, PAK1|1, and CCNB1. Moreover, in the known subcellular localization are detected all of them (Table S7). Additionally, SEC-MAP has also detected proteins with more than one localization; for example, in 4 subcellular localizations (Cyt, Mem, Org, Nuc), BCL-2 is detected by protocol #8 and 3 subcellular localizations (Cyt, Org, Nuc) by protocol #9 (Table S7).

Furthermore, analysis of subcellular protein localization by SEC-MAP has evaluated the effect of different protein extraction procedures. One example is RELA, which is detected in Cyt by protocol #7 and in the 4 subcellular fractions by protocol #8 (corroborating the reported subcellular localization and observed in the LC-MS/MS datasets). Another illustrative example is MAPK1, which is detected in the Cyt by protocols #7 and 8; however, protocol #9 detects it in Nuc localization. The observed subcellular localizations also correlate with LC-MS/MS datasets. (Table S7).

These results show: i. Protein extractions strategies are critical in the identified protein number by SEC-MAP. ii. Extraction strategy may be critical in the orthogonal integration with other omics datasets.

### 3.2. Deciphering Differential Protein Profiles by SEC-MAP

#### 3.2.1. Analysis of Intracellular Signaling Pathways by SEC-MAP 

**Figure 2:**
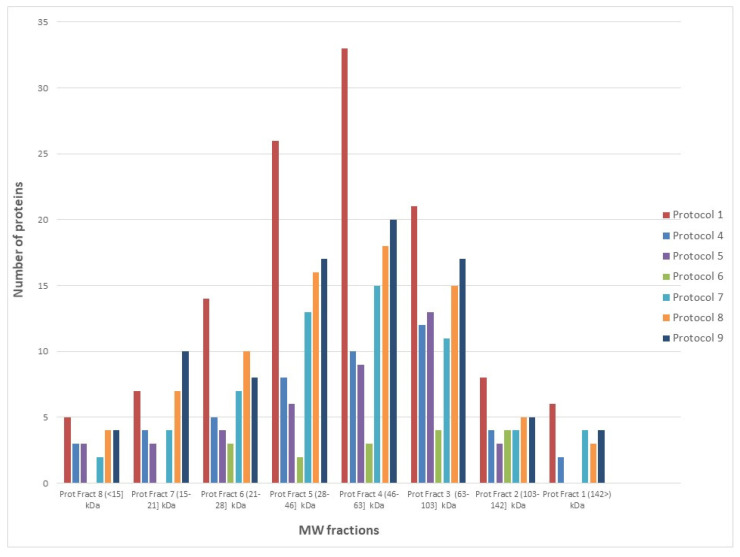
Effect of protein extraction protocols on protein identification by SEC-MAP approach.

This study integrates multiplex antibody microarray detections (MAP) with subcellular protein localization by SEC analysis. At first glance, a normal distribution of identified proteins (in all the studied protein extracts) is observed in the analyzed MW range (437-11 kDa) (Table S5 and Figure S5), where proteins are also detected in MW fractions that correspond with large protein sizes (Figure 2).

**Figure 3:**
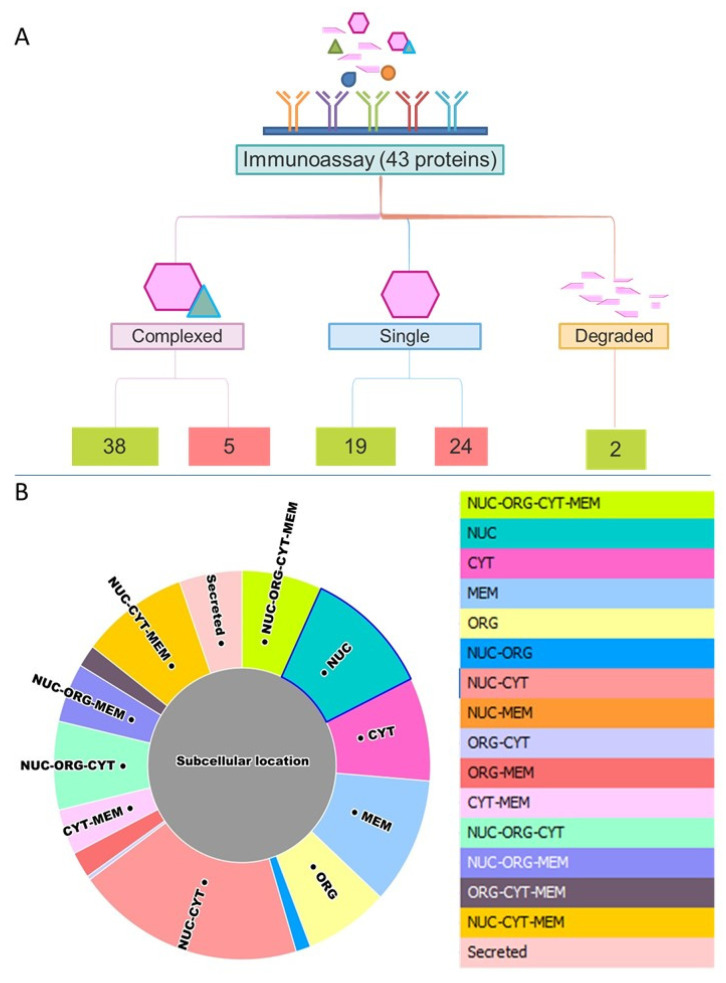
Summary of interest proteins selected for integration of SEC-MAP in the analysis of intracellular signaling pathways. (**A**): Schematic representation of proteins globally observed at different molecular isoforms (monomer, complexed, degraded). In green, the number of proteins detected, and in red, the number of non-detected proteins. (**B**): Summary pie chart of theoretical subcellular localization of proteins globally detected by all protein extraction protocols.

SEC-MAP approach allows the analysis of intracellular signaling pathways by simultaneous detection of multiple proteins. For this reason, several well-characterized signaling pathways are explored in the Ramos cell line: i. apoptosis regulation, ii. apoptosis inhibition, iii. BCR signaling pathway, iv. cell cycle control, v. STING pathway, vi. Damage-associated molecular patterns (DAMPs), vii. MAPK signaling, viii. senescence signaling [54,55,56,57]. In fact, SEC-MAP approach simultaneously detects heat shock proteins (HSP), Histones (3, 4), BCL-2, BAX, BAK1, BID, CASP7-8-9, IFN-γ, IL10, IL6, TNF-β, NF-Kβ1, TP53, RELA, MAP2K1, MAPK1, FOS, among others. Figure 3A shows the summary of SEC-MAP analysis of these proteins at the expected molecular weight (MW and/or expected SEC—fraction), as well as the detection of several proteins in other MWs than the reported one (Table S7). Moreover, the SEC-MAP approach allows us to analyze the signaling pathways based on protein localization (≥1 subcellular localization in one step by extraction protocols #7, #8, #9). Figure 3B shows a similar overall distribution for all SEC-MAP assays performed (Table S8).

#### 3.2.2. Multi-Protein Complex Analysis by SEC-MAP

**Figure 4:**
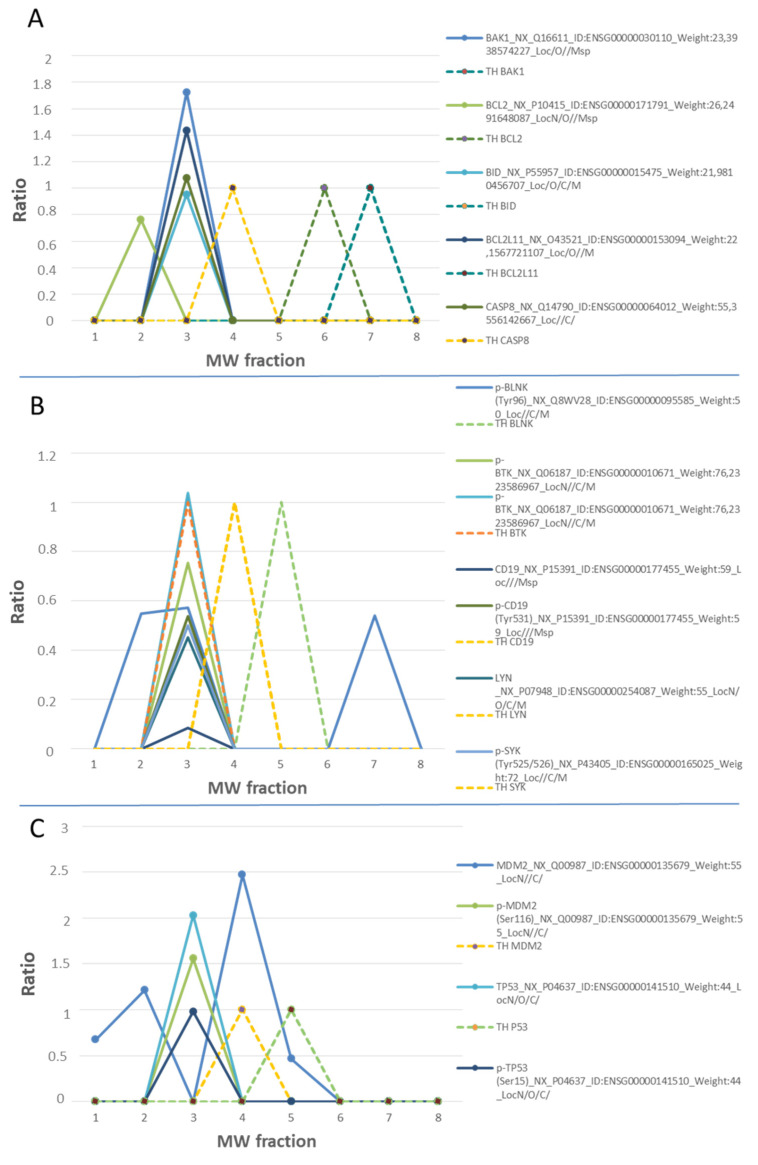
Multicomplex protein analysis by the SEC-MAP approach. Multiprotein complexes were detected at different protein extraction procedures. Peaks located at the same molecular weight (MW) fraction, and not found in their theoretical (TH) MW fraction, represent complexes formed by proteins when they are at a fraction above the expected one. When it is found in fractions below those expected, we speak of protein being hydrolyzed. If it is detected in theoretical fraction (dotted line), protein is found alone. (**A**). Proteins related to the BCL-2-BCL2L11 interaction signaling pathway. (**B**). Proteins related to the BTK pathway. (**C**). MDM2-TP53 interaction.

Nowadays, the inhibition of multi-protein complexes is one of the chemotherapeutical strategies in Burkitt’s lymphoma, such as Venetoclax, Nutlin-3, Ibrutinib work by inhibiting the formation of multi-protein complexes (Figure 4) [57,58,59]. In this study, several multi-protein complexes are analyzed by SEC-MAP. These include BCL-2, BCL2L11, BID, BAD, BAK1, CASP3, and CASP8, which have been detected as multi-protein complexes (Figure 4A and Supplementary Materials Dataset II).

**Figure 5:**
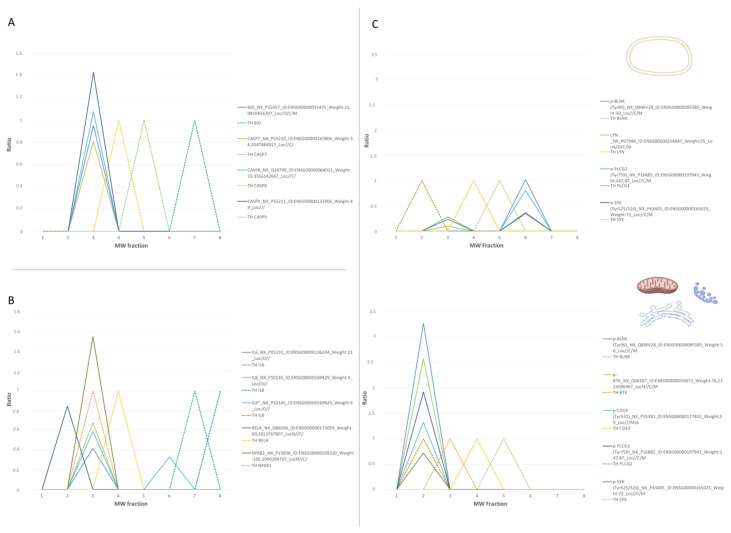
Multiprotein complex by SEC-MAP approach. Analysis of chosen multicomplexes involved in several cell signaling processes. (**A**): Proteins related to apoptosis inhibition. (**B**): Protein related to STING signaling. (**C**): Proteins related to B-cell receptor signaling in membrane and organelle subcellular localizations.

Moreover, the feasibility of SEC-MAP for the analysis of multi-protein complexes in only one intracellular signaling pathway. In Figure 4B, the BCR signaling pathway is analyzed by SEC-MAP, which reports protein complexes for PLCG2, SYK, CD19, ZAP70, LYN, BLNK, and BTK (Supplementary Materials Dataset II). Additionally, SEC-MAP analysis seems feasible to study protein complexes with discrimination at subcellular localization. A few illustrative examples are depicted in Figure 5A for apoptosis, Figure 5B for STING signaling and in Figure 5C for Ibrutinib targets (BTK, LYN, BLNK, ZAP70) at multiple subcellular localizations. Among these, SEC-MAP analysis also might be feasible to determine interacting partners, such as MDM2-TP53 (Figure 4C) and/or decipher novel protein complexes by the SEC-MAP analysis (Figure S6).

#### 3.2.3. Analysis of Specific Protein Isoforms and/or Variants by SEC-MAP

In the content of protein microarray, there are spotted antibodies targeting specific motifs/amino acid residues with post-translational modifications (PTMs) of the same proteins (see M&M sections and Supplementary Materials Dataset II). Moreover, several antibodies target the same protein (i.e., monoclonal -mAb-, polyclonal -pAb-). Hence, whether SEC-MAP analysis could use to evaluate the specificity and/or selectivity of antibodies against a particular protein, as well as to evaluate protein states (such as monomer, complexed, degraded) or to provide info about isoforms/variants of a particular protein (Table S9). Here, FOS protein could be a representative example, which is detected by one antibody in two subcellular localizations (Protocol #8: Org, Cyt), and in three subcellular localizations (Protocol #9: Mem, Org, Nuc) by a different antibody (Table S9). As proof of concept, a few protein isoforms are highlighted (in all cases, all antibodies used are in the M&M section): i. 4E-BP1 (clone 53H11) Rabbit mAb (as described in the M&M) and phospho-4E-BP1 (Thr37/46) (clon 236B4) Rabbit mAb. The rabbit mAb (clone 4E-BP1) allowed the protein identification in protocols #1, #4, #5, #7 (Cyt), #8 (Mem, Org and Cyt) and #9 (Mem, Org, Cyt and Nuc), while phospho-4E-BP1 (Thr37/46) is detecting the isoform only by protocol #1. ii.-STAT3 phospho-protein isoforms are detected SEC-MAP analysis by both antibodies (pAb-clone C-20-to STAT3 and p-STAT3 mAb-clone B-7-) with all the protein extraction protocols (#1 to #9). iii.-p38 isoforms: Several antibodies against phospho-isoforms of p38 (Phospho-p38 MAPK—Thr180/Tyr182-, (Rabbit mAb clone D3F9) the native form (Rabbit mAb clone 9212) detect the phosphorylated isoforms by SEC-MAP, allowing the determination of the relative abundance.

#### 3.2.4. Orthogonal Integration of SEC-MAP with Multi-Omics Datasets (RNA-Seq & LC-MS/MS)

**Figure 6:**
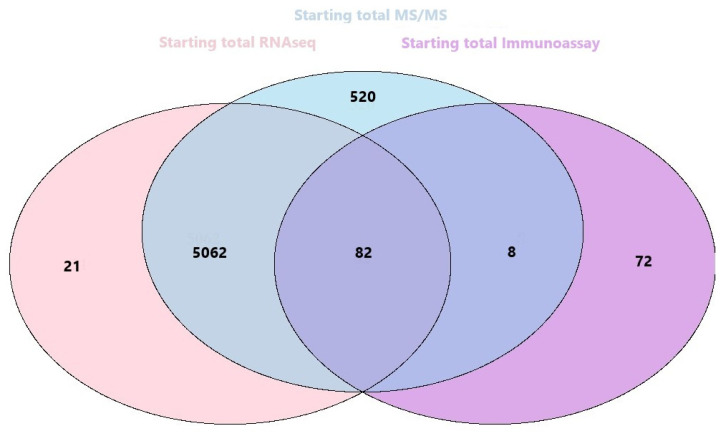
Venn diagram for orthogonal integration among all the datasets from SEC-MAP, LC-MS/MS, RNA-Seq (Number of proteins detected in each dataset).

Regarding the results of SEC-MAP integration with RNA-seq and LC-MS/MS datasets for Ramos cell line, a pipeline for orthogonal integration of datasets has been designed (Figure 6). First, a systematic database containing the global information from SEC-MAP analysis (global proteome, multi-protein complexes, specific protein isoforms/state, etc.) (Table S5) was required. Subsequently, correlations between the protein array content, RNA-seq and LC-MS/MS data were explored. Accordingly, 162 proteins are studied by SEC-MAP, RNA-seq, and LC-MS/MS, showing a strong inter-relationship between the proteins detected by these three omics strategies. Taking into account the orthogonal integration, first as the starting point, two-by-two correlations could be reported: i. 55.90% matching proteins between SEC-MAP and LC-MS/MS characterization; ii. 50.93% matching proteins between SEC-MAP and RNA-Seq information. iii. 99.59% matching proteins characterization between RNA-Seq and LC-MS/MS (see Table S10). Furthermore, according to neXtProt release 2020.01-17, 518 identified proteins belong to PE1, 6 to PE2, and 6 to PE5 of MS/MS information only and, 5121 belongs to PE1, 3 to PE2, and 3 to PE5 corresponding to the integration of MS/MS and RNA-Seq data (Table S11).

**Figure 7:**
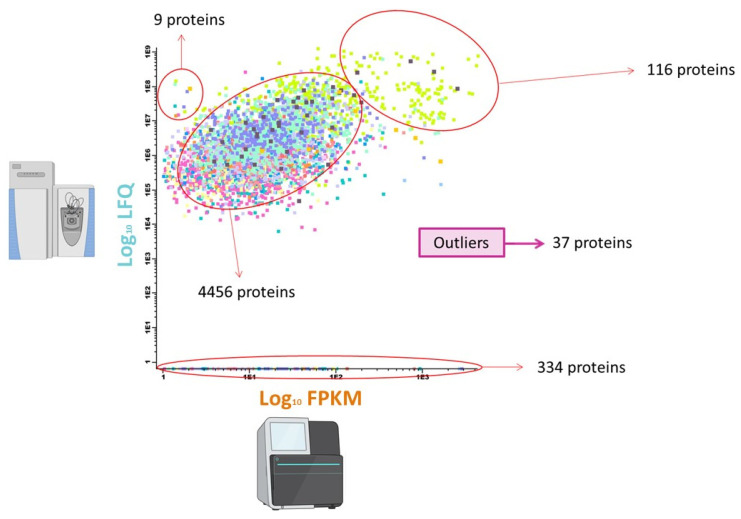
Dot-plot by Infinicyt 2.0 software displaying quantitative datasets for transcriptomics and LC-MS/MS characterization. Proteins/peptides are grouped according to different correlations between transcriptomics and proteomics datasets.

In this study, aiming to provide an approach suitable for globally graphical visualization of the orthogonal integration of multi-omics datasets (SEC-MAP, RNA-seq, LC-MS/MS), a bioinformatics tool—named Infinicyt (https://www.cytognos.com/infinicyt/2.0, accessed on 30 March 2019; Cytognos SL, Salamanca, Spain)—has been successfully implemented. This software tool also allowed to study a quantitative correlation between these datasets (i.e., FPKM and LFQ, for RNA-seq and LC-MS/MS respective) (Figure 7).

According to STRING and Reactome databases, it is observed that pathways related to the immune system and metabolism are significantly represented in 5 protein clusters. Metabolism of proteins and RNA pathways, cellular responses to external stimuli, transcription and DNA repair and cell cycle are represented in 4 proteins groups according to different high/low value log [LFQ] vs. high/low values of log [FPKM]. Finally, development biology and chromatin organization have been represented in three proteins groups according to log [LFQ] and log [FPKM] values (Table S12).

Bearing in mind similar patterns in the correlation between log[LFQ] and log[FPKM], the t-SNE plots provide insights from the functional point of view (within group of proteins): i. vesicle-mediated transport display high values on both parameters (Figure S7A), ii. organelle biogenesis and maintenance pathway displays high values in both parameters (Figure S7B); iii. hemostasis, DNA replication and programmed cell death for population averages appear as outliers on the correlation (Figure S7C,D). Deepening in this analysis by adding subcellular localization, several groups are observed on t-SNE plots according to the subcellular localization (Figure S7E–I, Table S12). For example, at Cyt localization, proteins related to cell–cell communication, programmed cell death, and hemostasis displayed low log[LFQ] and progressive log[FPKM] values. About proteins with ubiquitous localization in several subcellular localizations (Cyt + Mem + Nuc + Org) is observed as a homogeneous group at the t-SNE plot (with both high values, log[LFQ] & log [FPKM]) (Table S12).

## 4. Discussion

The synergistic integration of multi-omics datasets is highly dependent on multiple methodological aspects, which are also critical in the design and development of an algorithm for deepening the biological knowledge of intracellular signaling pathways. In this study, a simple approach has been designed and developed for the orthogonal integration of SEC-MAP, LC-MS/MS, and RNA-seq datasets corresponding to a Ramos cell line as a model. This orthogonal integration allowed—in a particular cellular situation—to decipher protein expression, isoforms, quantification at peptide and transcript levels, protein localization, protein interactions which also could be done at multiple cellular differentiation stages and/or physiological situations; thus, it could help uncover novel insights about the cellular dynamics and response to external stimulus.

Initially, sample preparation, as protein extraction procedures, has been revealed which is critical for orthogonal integration of multi-omics because of the expected influence in relative protein abundance, protein chemistry, protein stability, protein structure, tryptic digestion, protein solubility, and PTMs. It has been shown that protein extraction procedures have to be optimized for multi-omics integration because it could be an advantage/disadvantage in multi-pronged proteomics characterization (such as LC-MS/MS and SEC-MAP). Additionally, the specificity and selectivity of immunoassays are also affected by chemical labeling for detecting antigen-antibody interaction, which may hinder the epitopes and/or alter protein structure. Therefore, it is another critical factor for immunoassay validation of findings from RNA-seq and LC-MS/MS.

Overall, the importance of protein extraction procedures for further integration of proteomics with other omics datasets has been revealed. For example, protocols #2 and #3 have urea in the lysis buffer, which decreases the efficiency of biotin labeling and subsequent SEC-MAP analysis [60]. Another consideration is the chemical formulation of lysis buffers, such as the effect of non-ionic detergents (protocols #1, #5 and #6). Here, the role of critical micelle concentration (CMC) for each detergent is important in the protein extraction efficiency obtained for protocols #5 and #6. With this, it is shown that the detergent used in protocol #1 achieves a higher yield on protein extraction in comparison with other procedures. In this regard, among all protocols studied, protocol #1 is the one that gives us the best results; while protocol #6 produces the poorest results. Regarding subcellular localization, protocol #5 seems to be optimized for membrane protein extraction; meanwhile, for subcellular localizations, protocols #8 & #9 reported similar performance and effectiveness, with slight differences in membrane subcellular localization and organelle subcellular localization; which confirms the previously reported results by our group [19].

Regarding Human Proteome Project, it is a huge effort to detect “missing proteins”; which are classified into five groups (1–5) according to protein evidence (PE) [61]; then, protein enrichment is aligning a useful strategy to identify and detect “missing proteins” as it is shown in this study because a few proteins (reported as “missing proteins”) have been detected (mainly on group PE2 and PE5).

Recently, several studies employing SEC (in *Escherichia coli*, HEK293, osteosarcoma cells, liver human tissue and others) focused mainly on analysis of protein interactions (protein-protein, protein-metabolite, protein-small molecules), the difference between transcriptomics-proteomics levels or in combination with spatial proteomics [17,18,21,26]. In this study, SEC is combined with multiplex protein arrays (MAP) such that, not only it can identify single proteins, but also detect isoforms and protein complexes. Such use of SEC-MAP has also been reported by Kirtwodd et al., in 2013 [62]. In this study, it is observed that proteins are identified in a wide MW range by SEC-MAP; where protocol #1 seems to yield the best performance in most of the MW range studied; however, few other protocols give a better identification only for a few of the MW fractions, which might be related with the subcellular localization, presence of protein complexes, and inherent chemical properties of the proteins.

Regarding the SEC-MAP integration, many proteins (i.e., PAK1|P1, CCNB1, CASP, among others) are successfully detected at the expected MW and the subcellular localization as their confirmed existence by transcriptomics and MS/MS datasets. There is another group of proteins that are detected at the reported subcellular localization but at different MW, such as, BLC-2, RELA, MAPK1, MDM2, TP53, BLNK, SYK, CD19, LYN, ZAP70, PLCG2, suggesting the existence of protein–protein interactions and protein complexes. Thus, this approach is amenable for studying protein interaction networks and/or intracellular signaling dynamic as confirmed previously by our group in this study by correctly identifying the protein players in the previously reported pathways in this B lymphocyte cell line (such as senescence, evading growth suppressors, survival and death pathways, immune system evasion and immunoediting) [26,63,64,65]. This also opens the possibility to decipher interactions on newly reported pathways such as DAMPs (damage-associated molecular patterns), which might be useful for immunotherapies on lymphoma and leukemia because of their direct relation with the immunogenic cell death (ICD). Finally, similar to previously described by Díez P. et al. [19], it has been feasible to discriminate between protein groups in particular cell signaling pathways (such as immune system, metabolism, vesicle-mediated transport, organelle biogenesis and maintenance, homeostasis, etc.), monitoring subcellular localization and multi-omics correlation.

## 5. Conclusions

Multi-pronged proteomics characterization is highly dependent on protein extraction procedures, being a key step for functional outcomes from detection of proteins/multi-protein complexes in samples. Furthermore, it also reveals critical orthogonal integration of multi-omics data sets (such as SEC-MAP, LC-MS/MS, RNA-seq) and also provides complimentary info such as multi-protein complexes and subcellular localization. Moreover, the reported pipeline for multi-omics integration is useful for the HPP, as it systematically explores the compatibility of multi-omics data sets. Currently, the RNA-Seq technique provides us with information on the presence-absence of a particular protein of interest; meanwhile, MS/MS and SEC-MAP add data on protein localization, isoforms, and also, protein complexation status (complex-monomer-degraded). Consequently, SEC-MAP analysis seems to be a useful tool for orthogonal multi-omics integration, as it provides detailed complementary information about MW, subcellular localization, isoforms, PTMs, and protein complexes. Furthermore, SEC-MAP analysis seems feasible for determining predicted or unknown protein complexes and/or protein interactions in any sample (either from cell culture or clinical specimens).

With multi-omics orthogonal integration, it seems feasible to identify if a drug could target a protein complex of interest. It might help in the selection of drugs for a particular pathology.

## Figures and Tables

**Table 1:** Summary of protein extraction procedures.

					***Protocols***									
		***Buffer elements***			#1	#2	#3	#4	#5	#6	#7	#8	#9	
**Step 1**	*Phosphatase Inhibitor (mM)*	***TCEP***			1	1	1	1	1	1	1	1	1	1
		***PMSF***			1	1	1	1	1	1	1	1	1	1
		***NaF***			1	1	1	1	1	1	1	1	1	1
		***Sodium orthovanadate***			1	1	1	1	1	1	1	1	1	1
		***β-glycerophosphate***			1	1	1	1	1	1	1	1	1	1
		***Sodium pyrophosphate tetrabasic decahydrate***			1	1	1	1	1	1	1	1	1	1
	*Salt (mM)*	***NaCl***			140	-	-	140	-	-	140	-	-	400
		***KCl***			-	-	-	-	15	15	-	15	15	-
	*Nuclear envelope protector (mM)*	***MgCl2***			-	-	-	10	2	2	10	2	2	2
	*Metalloproteinase inhibitor (mM)*	***EDTA***			50	-	-	-	1	1	-	1	1	1
	*Chaotropic agent (M)*	***Urea***			-	9	7	-	-	-	-	-	-	-
		***Thiourea***			-	-	2	-	-	-	-	-	-	-
	*Buffer solution (mM)*	***Tris/HCl***			20	-	30	-	-	-	-	-	-	-
		***HEPES***				20	-	5	30	30	5	30	30	30
	*Thickening agent (%-v/v-)*	***Glycerol***			10	-	-	-	20	20	-	20	20	-
	*Non ionic detergent (%-v/v-)*	***IGEPAL***			1	-	-	-	-	-	-	-	-	-
		***Tween 20***			-	-	-	0.1	-	-	0.1	-	-	-
		***Laurylmaltoside***			-	-	-	-	10	-	-	-	-	-
		***Triton X-100***			-	-	-	-	-	1.5	-	-	-	-
					***Protocols***									
		***Buffer elements***			#1	#2	#3	#4	#5	#6	#7	#8	#9	
**Step 1**	*Non ionic detergent (%-v/v-)*	***Digitonin***			-	-	-	-	-	-	-	0.015	0.015	-
		***Octyl-β-D-glucopiranoside***			-	-	-	-	-	-	38	-	-	-
**Step 2**	*Non ionic detergent (%-v/v-)*	***Tween 20***			-	-	-	-	-	-	-	5	5	-
**Step 3**	*Salt (mM)*	***NaCl***			-	-	-	-	-	-	-	14	-	-
	*Non ionic detergent (%-v/v-)*	***IGEPAL***			-	-	-	-	-	-	-	-	-	0.5
**Step 4**		***Laurylmaltoside***			-	-	-	-	-	-	-	1	-	1
**Centrifugation**		***Time (min)***			15	15	15	15	5	5	15	5	5	5
		***10^3^***× ***g***			15	15	12	12	16	16	15	0.5	0.5	3/15
***Legend***														

	Phosphatase Inhibitor		Salt		Nuclear envelope protector						Metalloproteinase inhibitor			

	Chaotropic agent		Buffer solution		Thickening agent						Non ionic detergent			

			Centrifugation											

## Data Availability

MS/MS data are available via ProteomeXchange with identifier PXD003939 at EBI-EMBL.

## Supplementary Materials

The following are available online at https://www.mdpi.com/article/10.3390/biom11121776/s1. Figure S1: Comparison of efficiencies according to the extraction protein processing used. Panel A: Amount protein obtained. Panel B: SDS-PAGE 12% Coomassie blue and silver staining. Figure S2: Comparison of efficiencies in differing concentrations of biotin. Panel A: Number of spots detected for the fraction. Panel B: Number of spots not detected for fraction. Figure S3: Graphic of molecular weight markers used after performing fast protein liquid chromatography (FPLC) technique. Figure S4: Descriptive analysis of behavior for each extraction protocol. Panel A: Global number of spots detected. Panel B: Number of spots detected for each subcellular fraction by four fraction protocol. Figure S5: Test of normality for protein extraction protocols. Panel A: Protocol 1. Panel B: Protocol 4. Panel C: Protocol 5. Panel D: Protocol 6. Panel E: Protocol 7. Panel F: Protocol 8. Panel G: Protocol 9. Figure S6: Multicomplex analysis for peaks by protocol 1. Panel A: Interaction detected in Fraction 2. Panel B: Interaction detected in Fraction 3. Panel C: Interaction detected in Fraction 2 and 4. Figure S7: Functional interaction (Reactome) and t-SNE plots (TS) of Infinicyt for groups obtained after analyses by values quantitative LFQ and FPKM. Panel A: Low LFQ and Progressive FPKM in Cyt for vesicle-mediated transport. Panel B: High LFQ and FPKM in Org for Organelle biogenesis and maintenance. Panel C: Outlier with High LFQ and Medium FPKM value (Hemostasis and Extracellular matrix organization). Panel D: Outlier with High LFQ and High FPKM value (Hemostasis). Panel E: Low LFQ and Progressive FPKM in Cyt. Panel F: Low LFQ and Progressive FPKM in Mem. Panel G: Low LFQ and Progressive FPKM in Org. Panel H: Low LFQ and Progressive FPKM in Nuc & Org. Panel I: Low LFQ and Progressive FPKM in Nuc & Cyt. Panel J: Low LFQ and Progressive FPKM in Org & Cyt. Table S1. Designer of subarray printing (Antibodies and controls—green: positive controls; and yellow: negative controls-). Table S2: List of protein target included in protein microarray. Table S3: Mapping of proteins with unique peptide. Table S4: Mapping of proteins with unique peptide and the highest FPKM value. Table S5: Descriptive statistic and summary information about protein extraction protocols by immunoassay and integration with RNA-Seq and MS/MS. Table S6: Z values results of comparisons between protein extraction protocols, both globally and by molecular weight fraction. Table S7: Deep analysis about state of protein, theoretical and assay subcellular localization (MS/MS and microarray) and RNA-Seq information of selection proteins according to relevance and interesting reported in literature. Table S8: Number of proteins detected in each subcellular localization and possible combinations. Table S9: Proteins detected with different antibodies and post-translational modification (PTM) detection. Table S10: Percentage two to two relationship for MS/MS, RNA-Seq and immunoassay techniques, Table S11: Orthogonal integration and missing proteins detected. Table S12: Functional interaction (Reactome) and biological enrichment processes (KEGG) present in groups obtained after analyses and visualization in Infinicyt by values quantitative LFQ and FPKM. Supplementary Materials Dataset I: Visualization of integration information from transcriptomic, proteomic and immunoassay. Supplementary Materials Dataset II: Dataset Immunoassay, proteomic and transcriptomic data integration.
