# Supplementary figures and images for: Deepening into Intracellular Signaling Landscape through Integrative Spatial Proteomics and Transcriptomics in a Lymphoma Model
Source: Biomolecules. 2021 Nov 26;11(12):1776. doi: 10.3390/biom11121776 (PMC8699084; doi:10.3390/biom11121776)

A

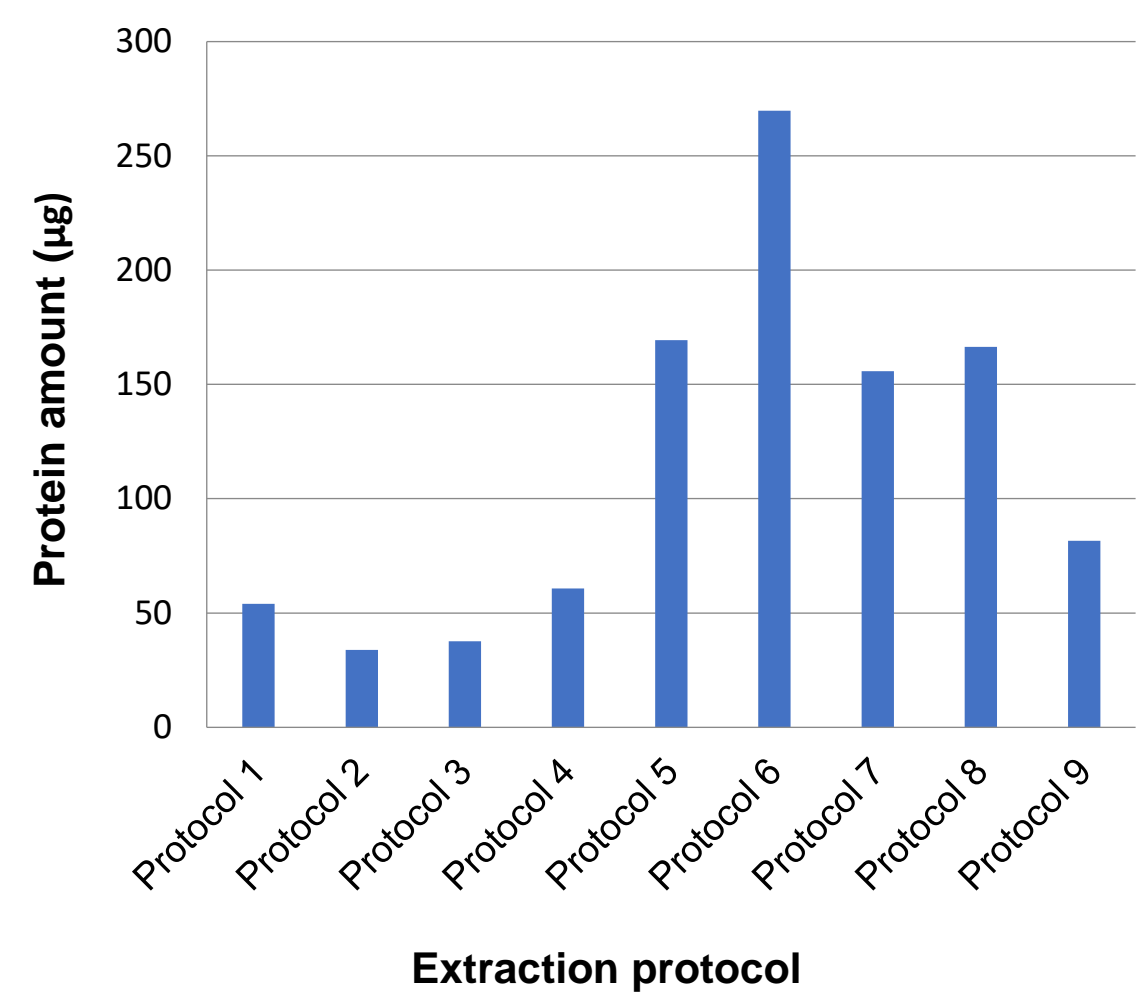

B

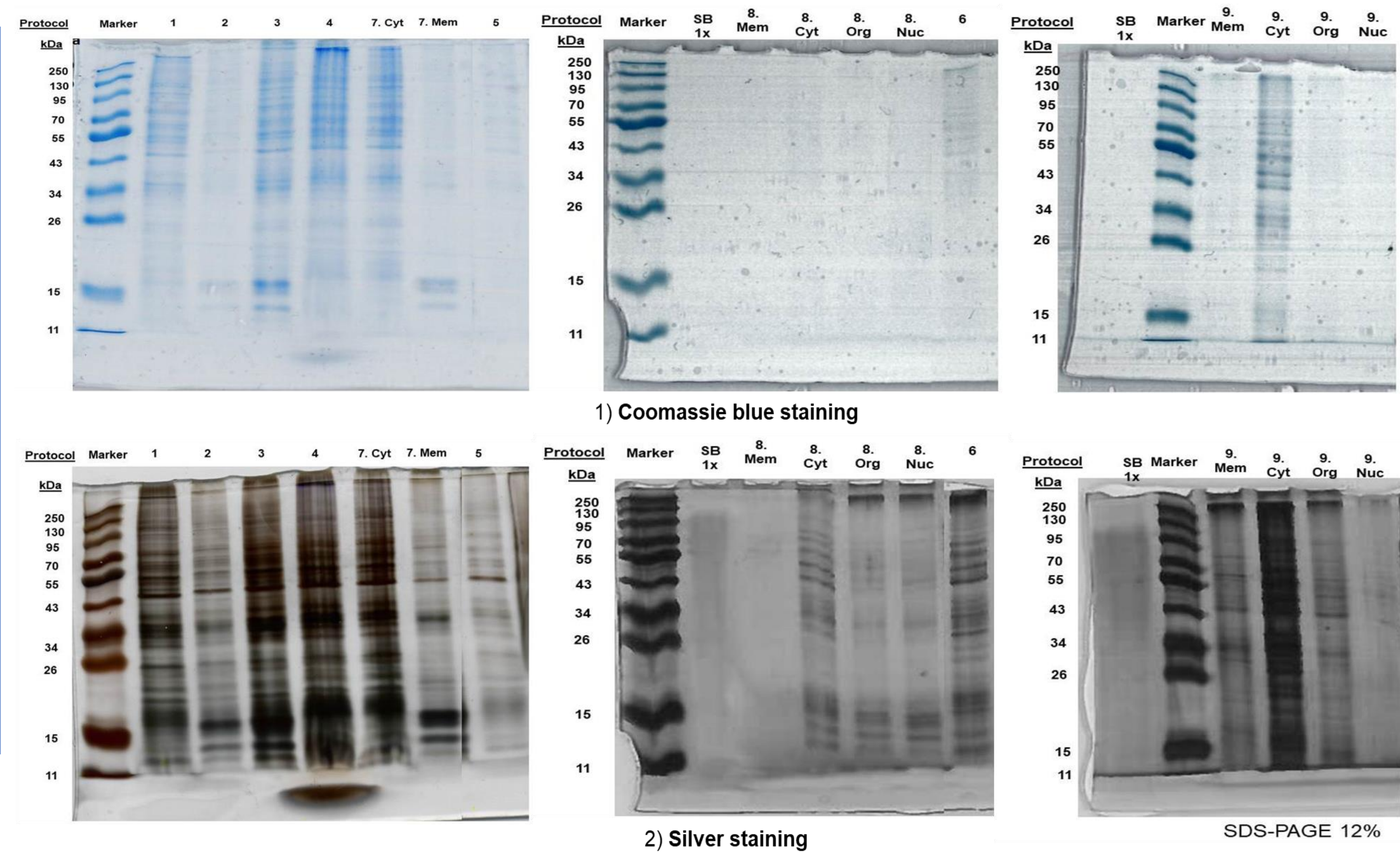

Supplement: Supplementary file 1 [file biomolecules-11-01776-s001.zip › Figure S1.pdf]

A

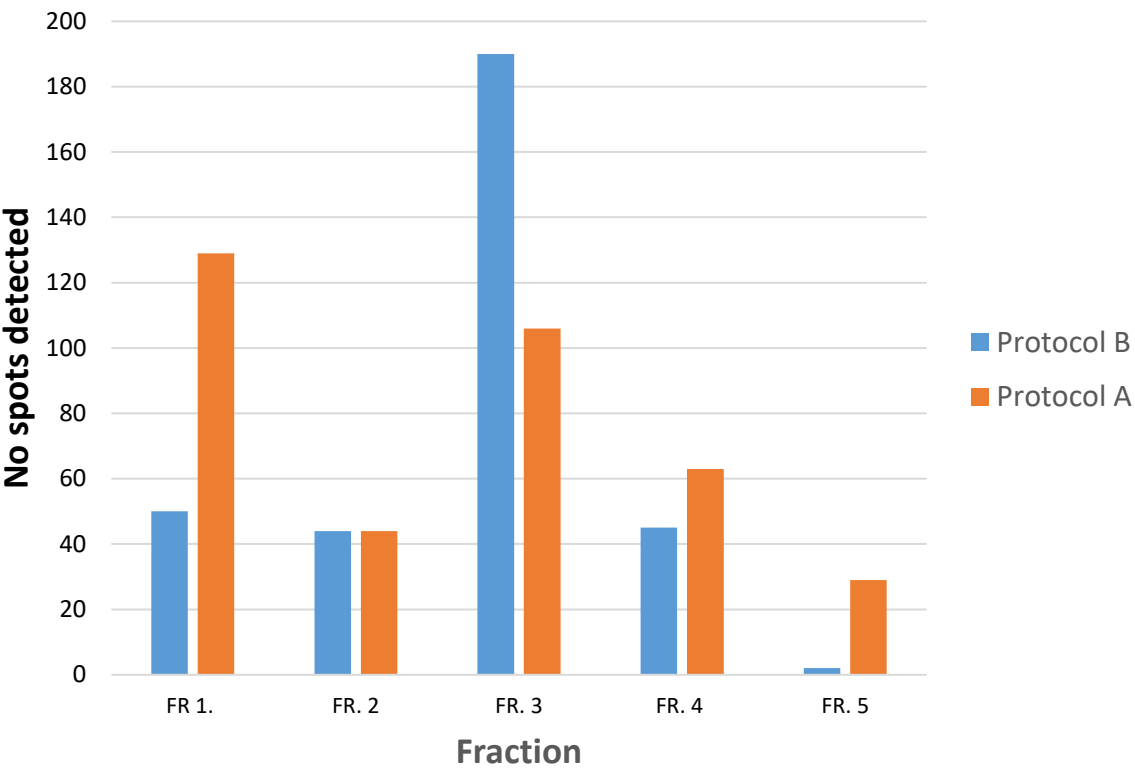

B

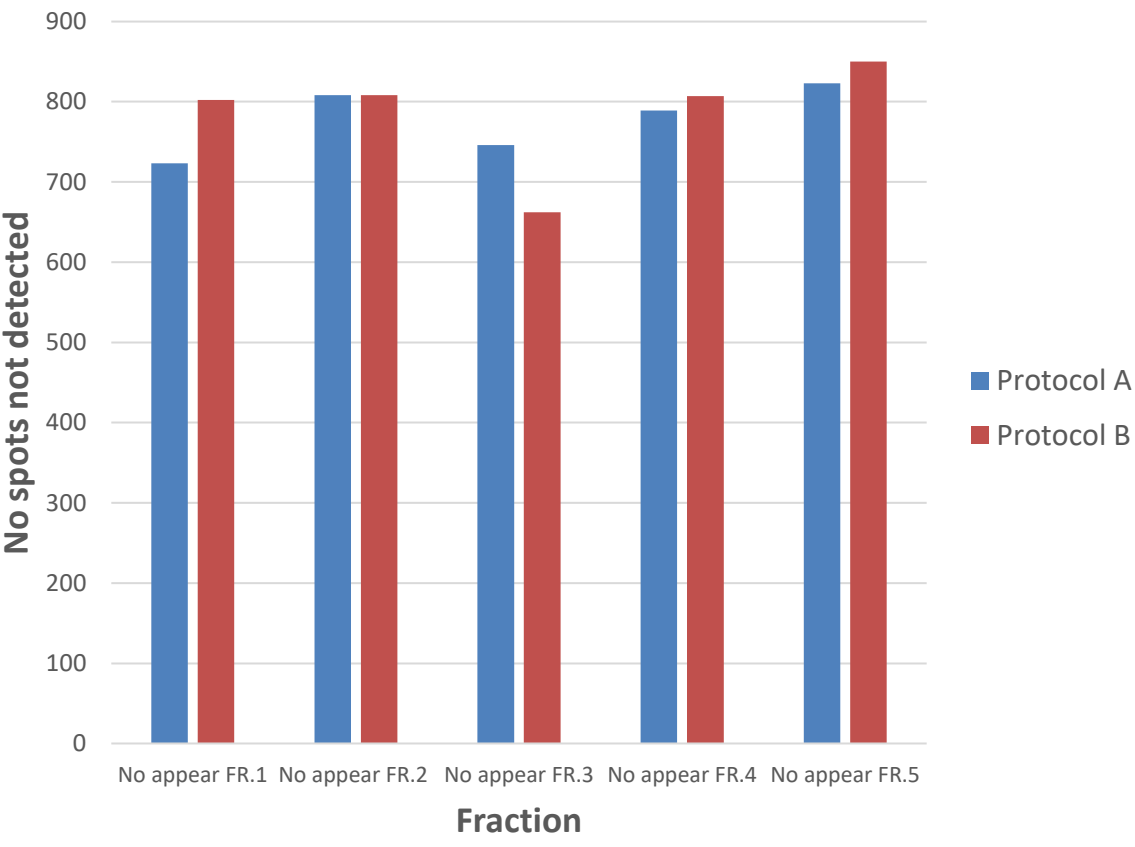

Supplement: Supplementary file 1 [file biomolecules-11-01776-s001.zip › Figure S2.pdf]

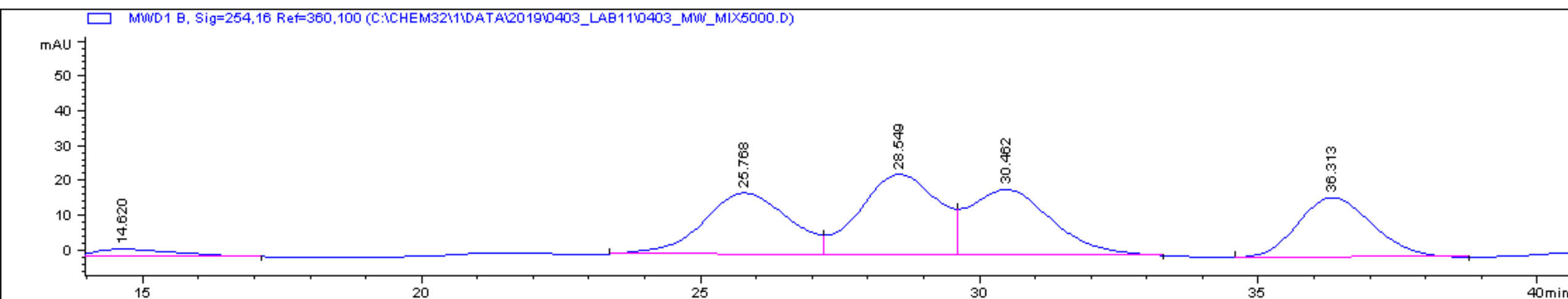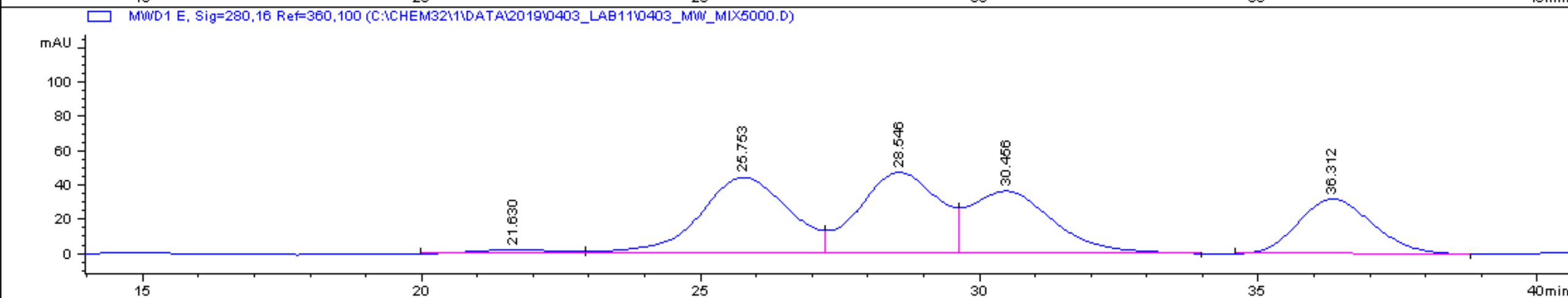

Supplement: Supplementary file 1 [file biomolecules-11-01776-s001.zip › Figure S3.pdf]

**A**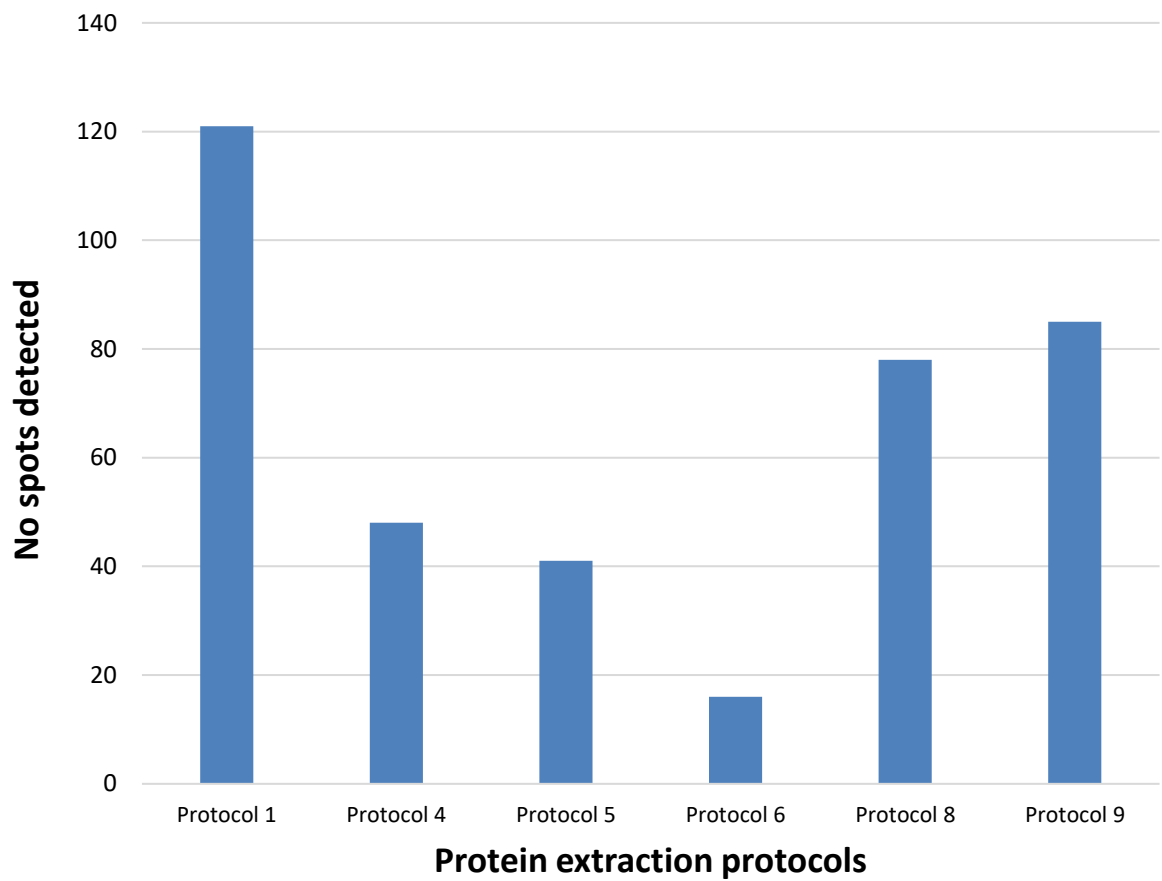**B**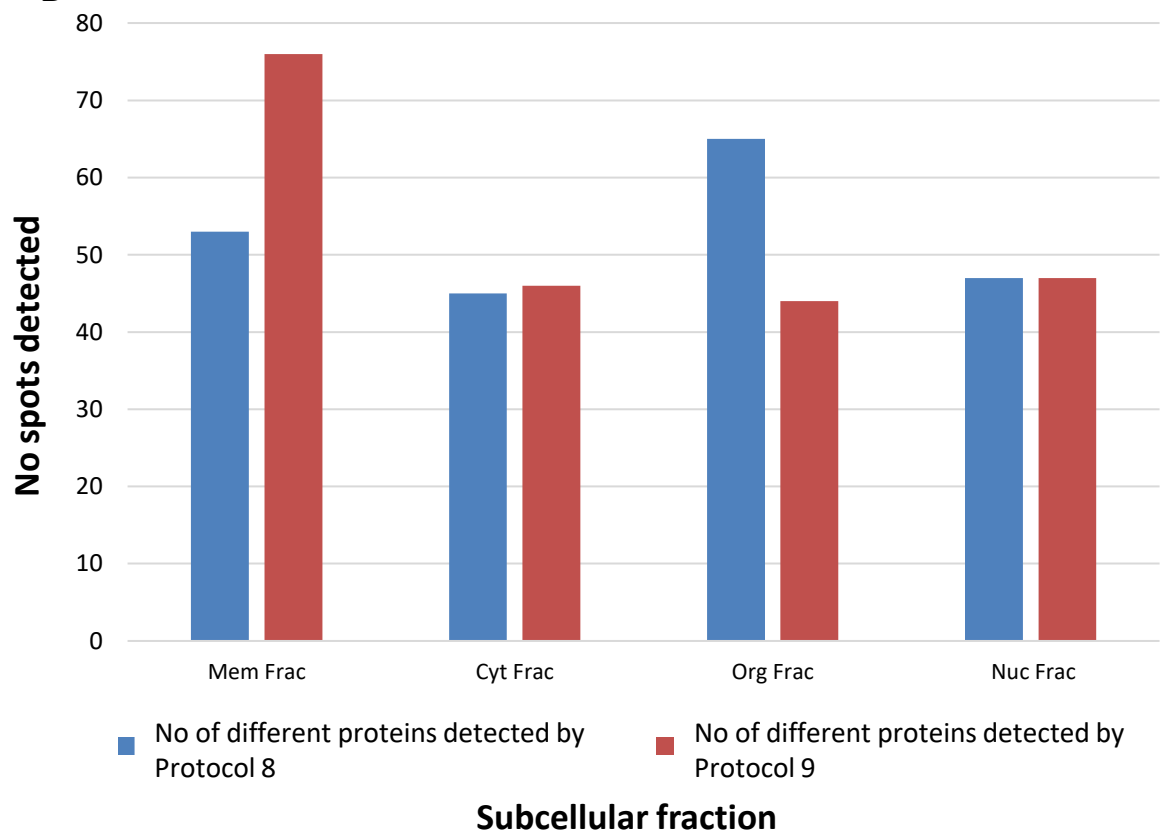

Supplement: Supplementary file 1 [file biomolecules-11-01776-s001.zip › Figure S4.pdf]

**A**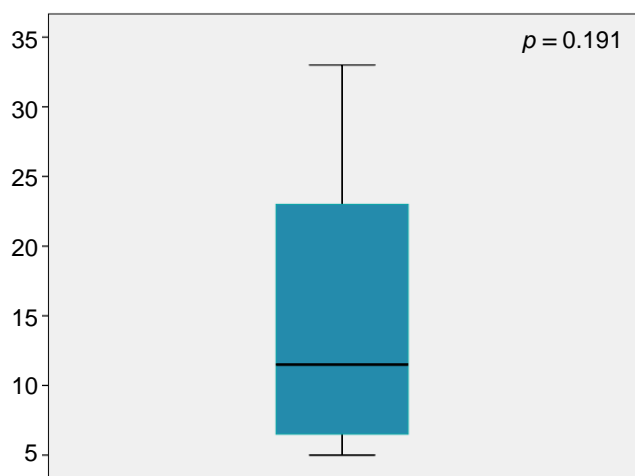**B**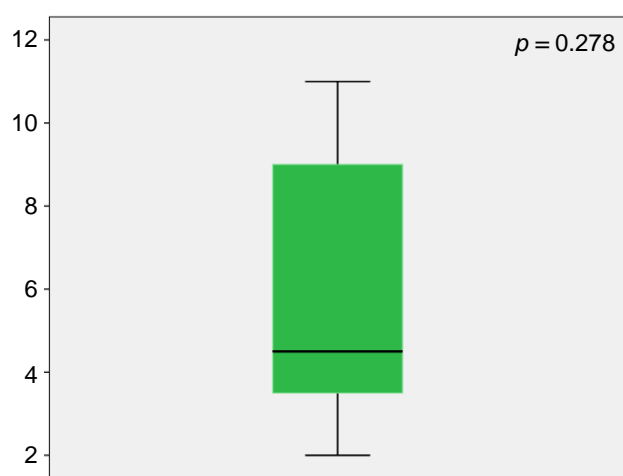**C**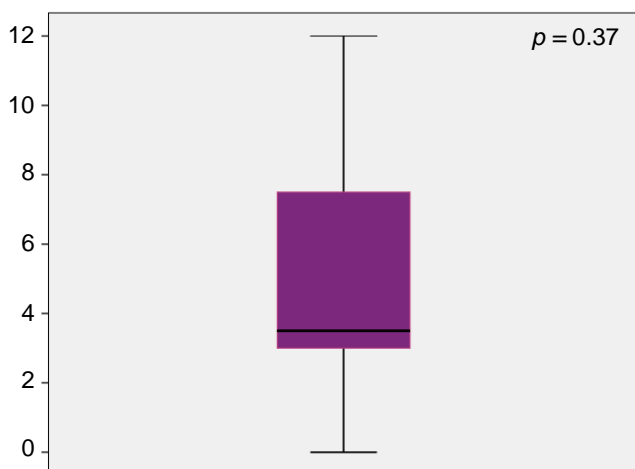**D**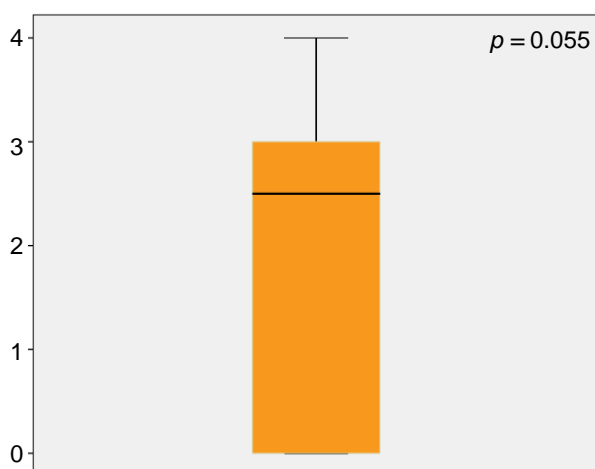**E**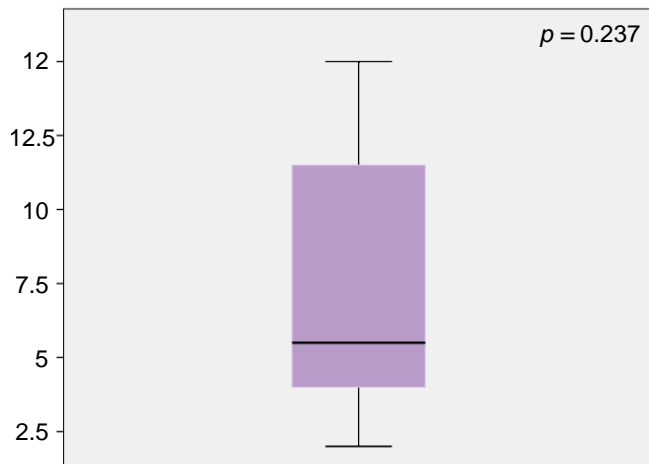**F**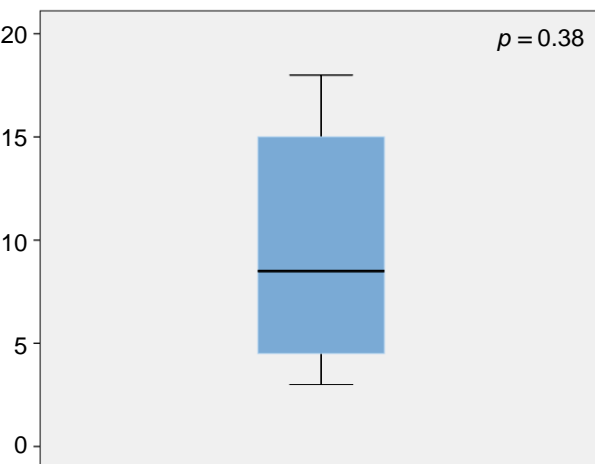**G**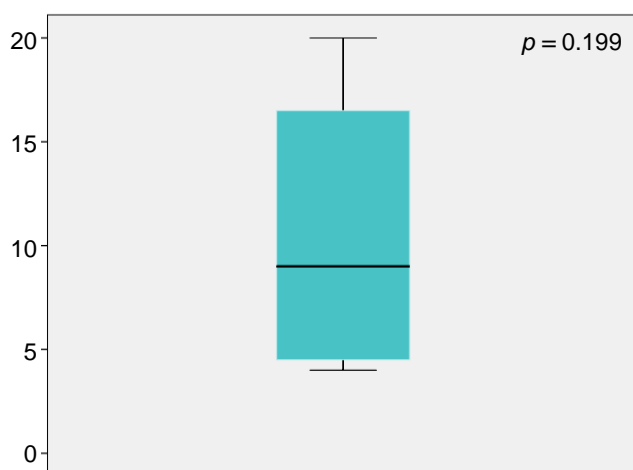

Supplement: Supplementary file 1 [file biomolecules-11-01776-s001.zip › Figure S5.pdf]

A

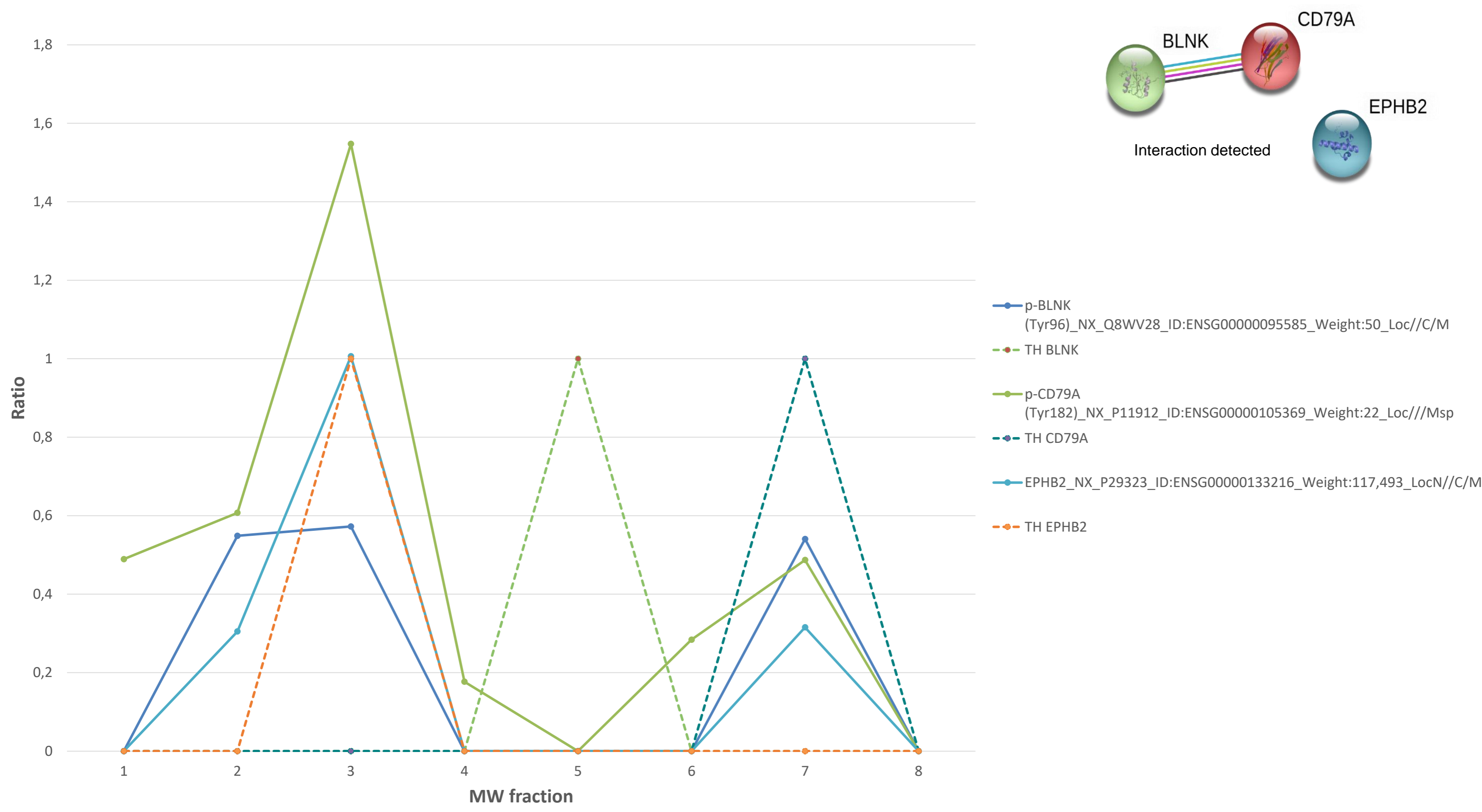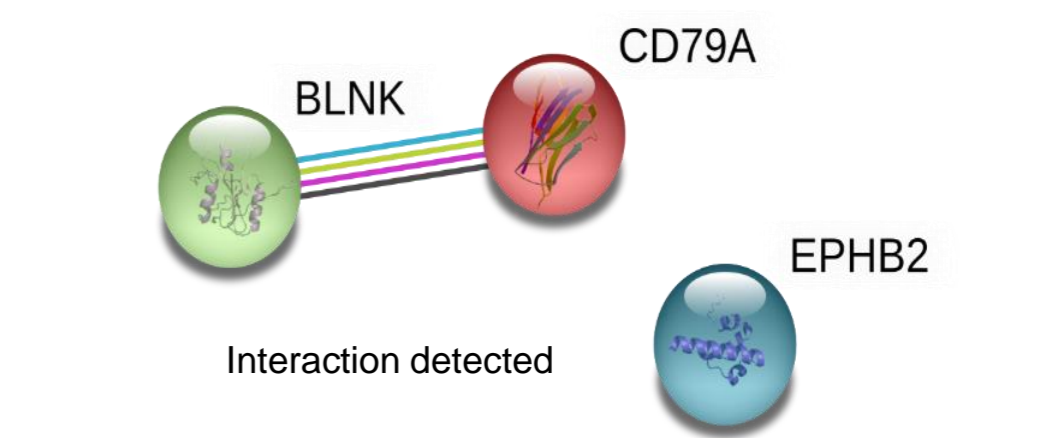

B

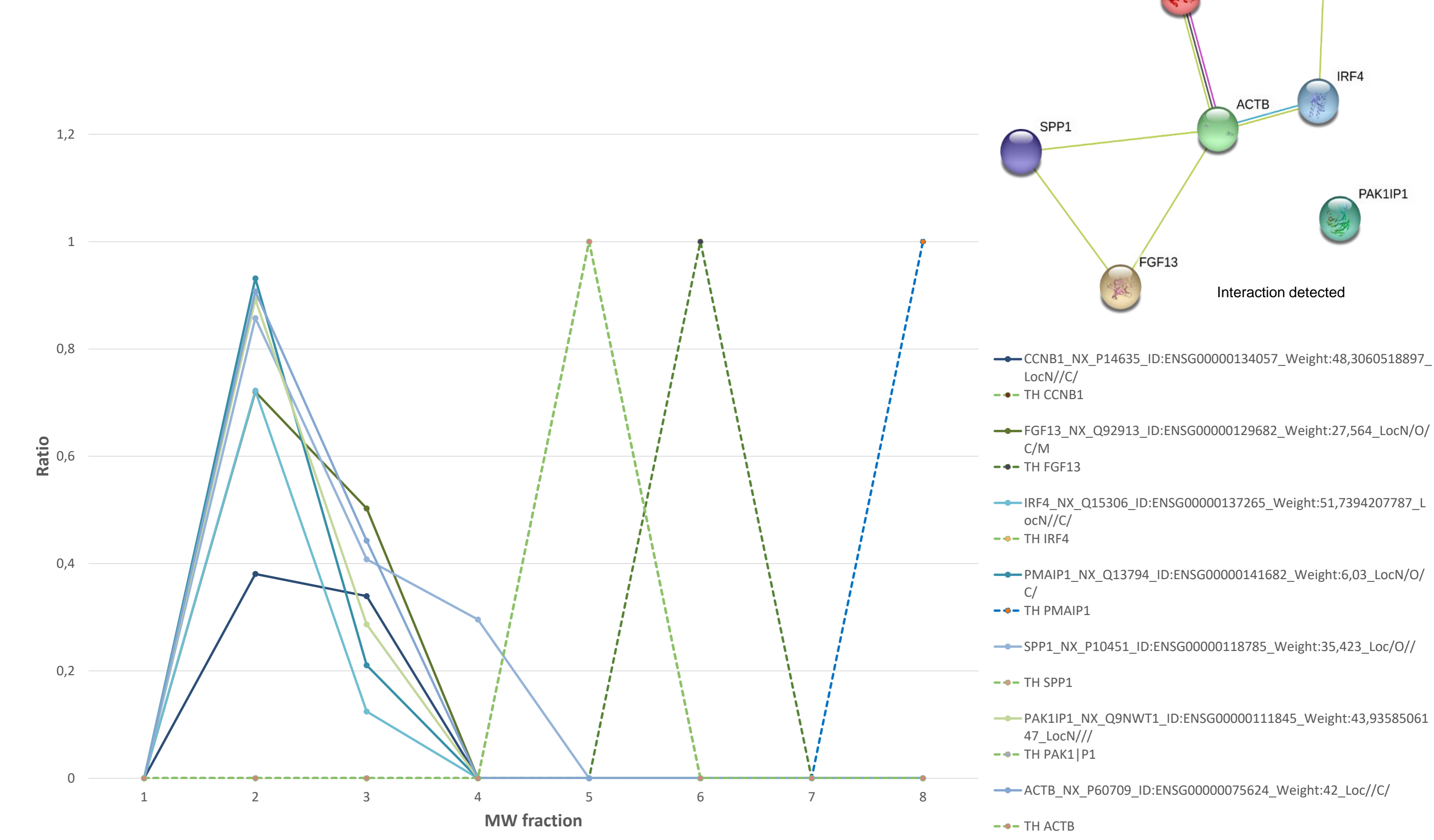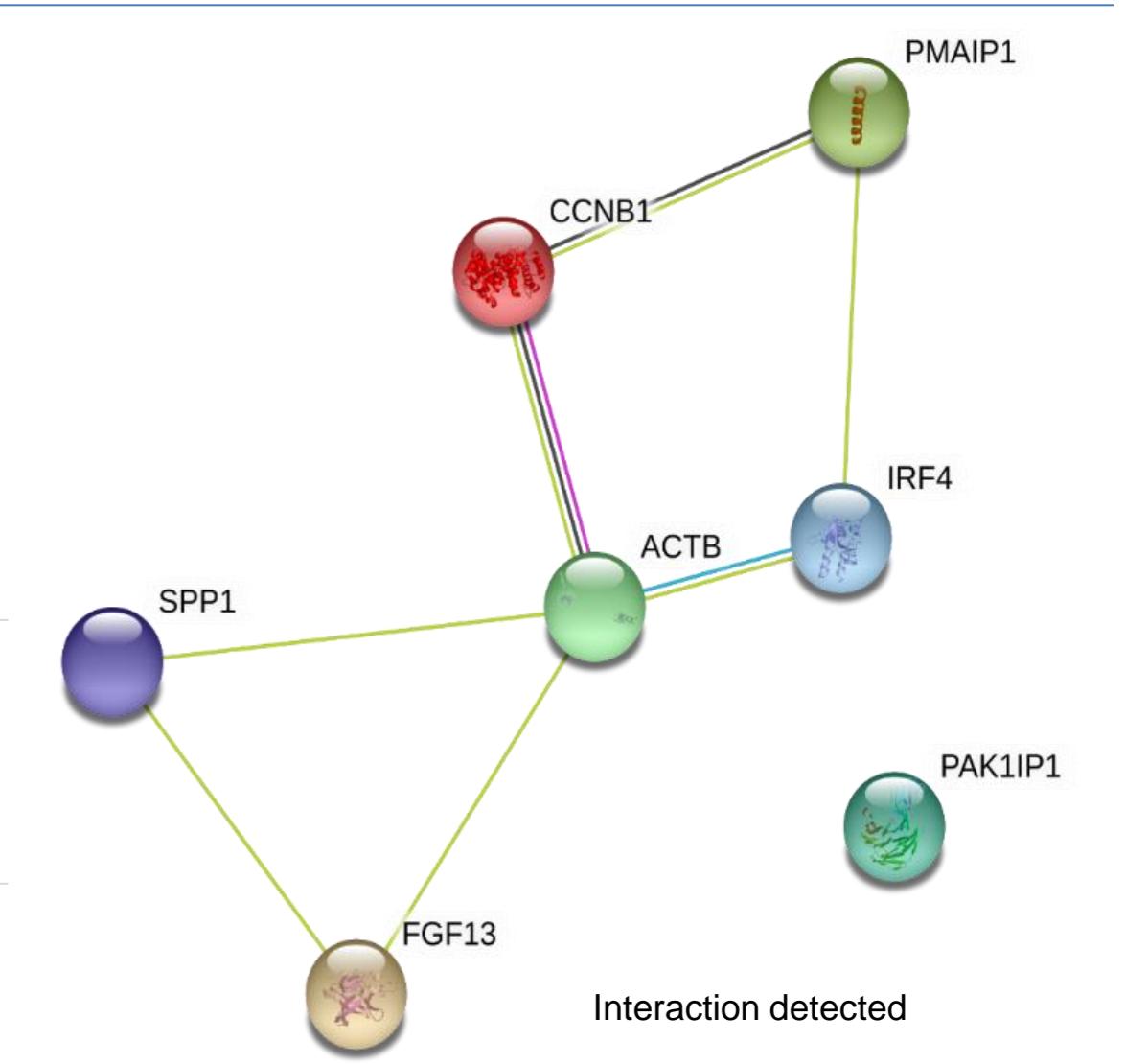

C

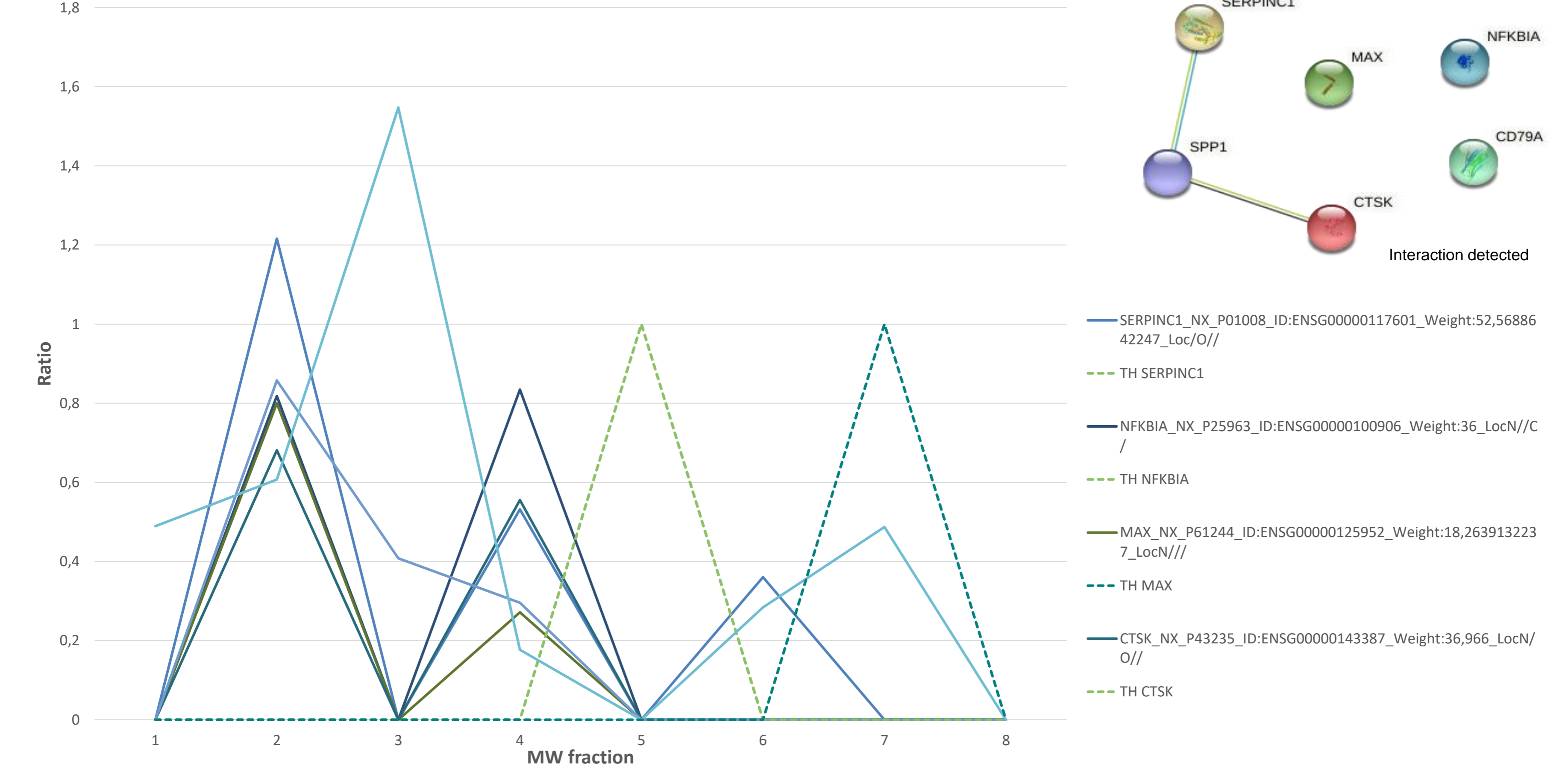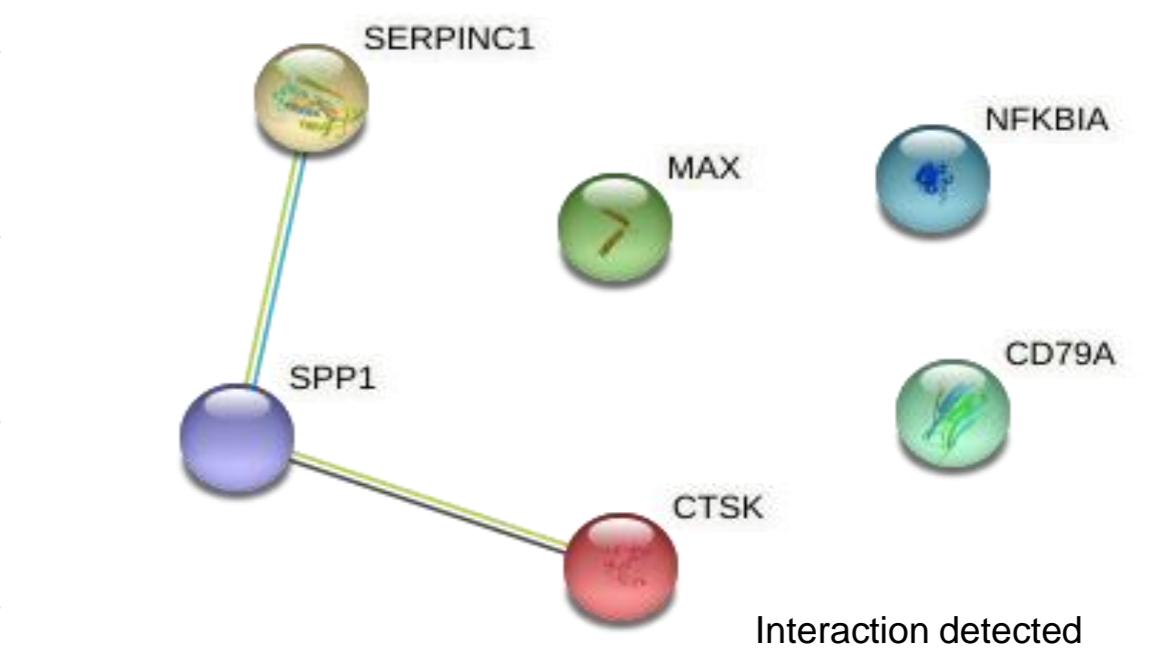

Supplement: Supplementary file 1 [file biomolecules-11-01776-s001.zip › Figure S6.pdf]

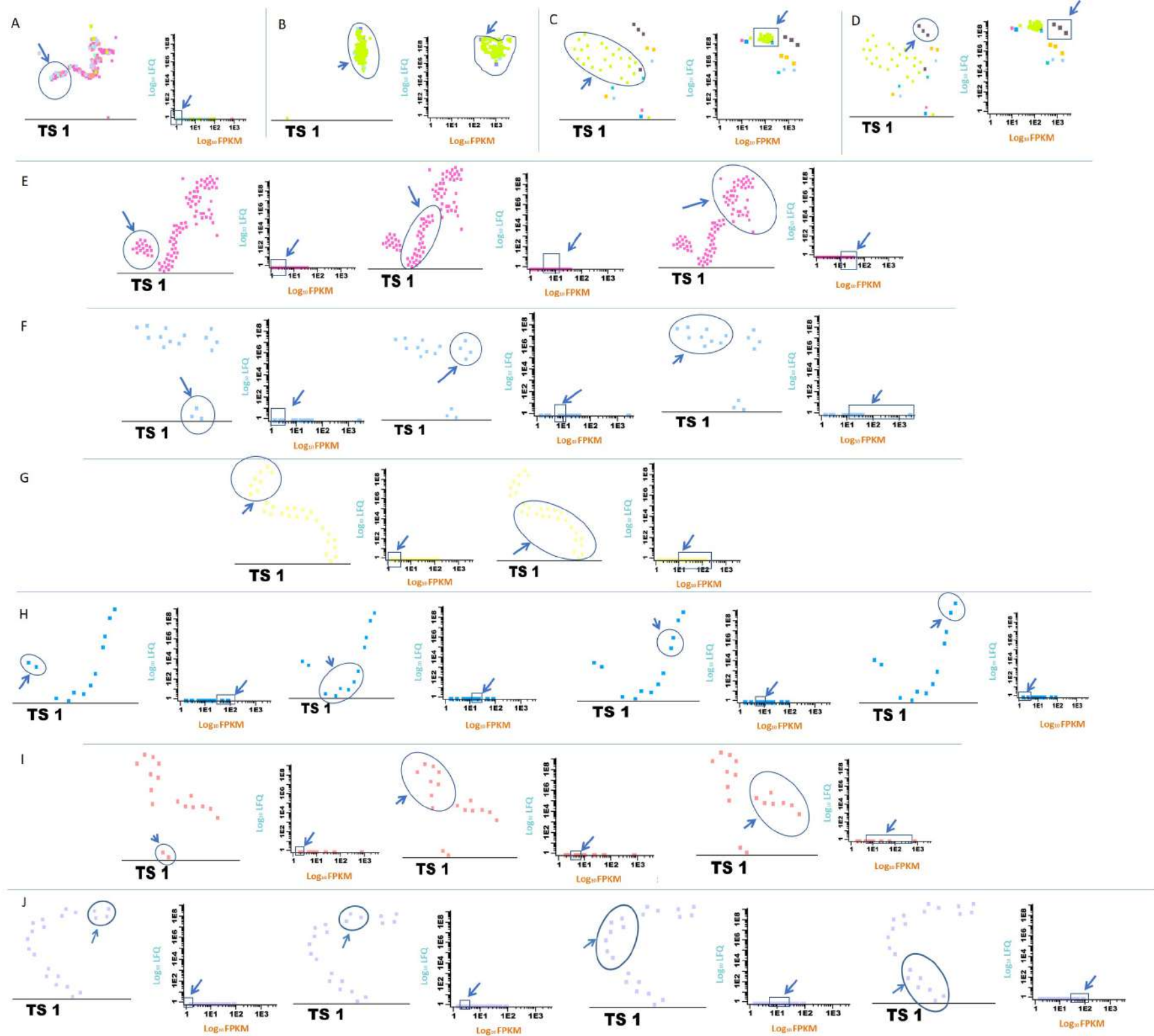

Supplement: Supplementary file 1 [file biomolecules-11-01776-s001.zip › Figure S7.pdf]
